# Large-scale pyrodiversity is not needed to beget ant diversity in an Australian tropical savanna

**DOI:** 10.1007/s00442-025-05683-7

**Published:** 2025-02-28

**Authors:** François Brassard, Brett P. Murphy, Simon Ferrier, Alan N. Andersen

**Affiliations:** 1 Research Institute for the Environment and Livelihoods, Charles Darwin University, Ellengowan Drive, Casuarina, Darwin, NT 0810 Australia; 2 Commonwealth Scientific and Industrial Research Organisation, GPO Box 1700, Canberra, ACT 2601 Australia

**Keywords:** Pyrodiversity, Reserve selection, Fire disturbance, Formicidae, Savanna

## Abstract

The hypothesis that pyrodiversity begets biodiversity is foundational to conservation management in fire-prone ecosystems and has received extensive research attention. However, empirical evidence for the hypothesis remains ambivalent. Moreover, few studies directly assess the key question of how much pyrodiversity is needed to conserve all species within a community. A novel way of addressing this is to use the biodiversity–maximisation approach developed for reserve selection as part of strategic conservation planning. We apply this approach to an ant dataset from a long-term fire experiment in northern Australia to establish how many of the six experimental fire treatments are required to represent all local ant diversity. We identified the treatment combinations required to maximise species richness and geometric mean abundance. We repeated this for six fire-activity classes based on cumulative fire intensity experienced by plots over the course of the experiment. We found that a very limited number of fire treatments or fire activity classes were needed to represent all of the highly diverse ant species and to maximise the geometric mean abundance of ants. We attribute this to the substantial small-scale heterogeneity of fire behaviour and vegetation structure within individual fire treatments. We conclude that high pyrodiversity at larger spatial scales is not required for sustaining ant biodiversity in our study system. We believe that a reserve selection approach is a powerful method for assessing how much pyrodiversity is needed to conserve biodiversity and recommend that it be applied to other taxa and other ecosystems.

**Supplementary Information:**

The online version contains supplementary material available at 10.1007/s00442-025-05683-7.

## Introduction

The concept that “pyrodiversity begets biodiversity” was introduced three decades ago (Martin and Sapsis 1992) and has become a foundation for the use of fire for biodiversity conservation in flammable biomes (Bowman et al. 2016; Kelly and Brotons 2017; Jones and Tingley 2022). Pyrodiversity—spatially and/or temporally variable fire regime attributes, such as fire frequency, seasonality, and severity—is hypothesised to promote biodiversity because it increases environmental heterogeneity at the landscape scale (He et al. 2019). Although this is intuitively appealing, a more critical consideration of the levels of pyrodiversity needed for maintaining biodiversity is required (Parr and Andersen 2006). Over the past three decades, numerous studies examined the pyrodiversity–biodiversity hypothesis for fauna across a range of biogeographic regions and taxa, assessing faunal responses to spatial and temporal variation in fire attributes ((Jones and Tingley 2022), Appendix, Table S1). No consistent patterns have emerged (Jones and Tingley 2022), with only about half of the studies (48%, 22 of 46) providing support for the hypothesis (Appendix, Table S1). Although such ‘vote counting’ is imperfect as it does not consider effect sizes, uncertainties and the various study designs, the lack of consistency is perhaps not unexpected. This is because the relationship between pyrodiversity and biodiversity is context-dependent (Kelly and Brotons 2017), such that assessments of different pyrodiversity and biodiversity metrics on different taxa across bioregions is unlikely to produce identical results.

Most studies addressing the pyrodiversity–biodiversity hypothesis have focussed on documenting differences between species in their responses to fire (Kelly et al. 2015; Davies et al. 2018; Jolly et al. 2022), or in describing the relationships between various measures of pyrodiversity and biodiversity (Docherty et al. 2020; Ulyshen et al. 2022). However, this does not directly address the key management question of how much pyrodiversity is needed to maintain biodiversity. Not all pyrodiversity necessarily has conservation benefit and indeed some is likely to have a negative impact (Parr and Andersen 2006). Moreover, fires can be highly patchy in their behaviour and so any individual fire may be inherently pyrodiverse at multiple spatial scales (Brassard et al. 2023). For conservation management, the key question is not whether a relationship between pyrodiversity and biodiversity exists, but the extent to which pyrodiversity needs to be actively managed to conserve biodiversity. It is plausible that a very limited range of fire regimes will adequately conserve biodiversity without the need for managers to create a complex mosaic of different fire regimes (Parr and Andersen 2006; Andersen 2021). However, answering this question requires direct assessments of how much pyrodiversity is needed to conserve all species within a landscape. This is rarely advocated for, even in recent exhaustive reviews of pyrodiversity research (Jones and Tingley 2022; Steel et al. 2023).

A novel way of addressing the question of how much pyrodiversity is required to maintain biodiversity is to use the biodiversity–maximisation approach developed for reserve selection as part of strategic conservation planning (see examples in Csuti et al. 1997; Briers 2002; Sarkar 2012). Such an approach typically uses optimisation algorithms to identify the number and combination of conservation reserves needed to represent biodiversity, based on known habitat associations of species. Here we use such an approach, based on associations with fire rather than habitat, to assess how much pyrodiversity is needed to support ant diversity in an Australian tropical savanna.

Tropical savannas are the world’s most fire-prone biome (Van Der Werf et al. 2017), and are highly biodiverse, in many ways comparable to that of tropical rainforests (Murphy et al. 2016). Ants are an ecologically dominant faunal group globally, playing key roles as soil engineers, seed dispersers, nutrient cyclers, predators and prey (Folgarait 1998; Lach et al. 2010; Lengyel et al. 2010; Del Toro et al. 2012). As such, ants are frequently used by land managers as bioindicators of ecological change (Andersen and Majer 2004; Underwood and Fisher 2006). Ants are particularly abundant in tropical savannas (Schultheiss et al. 2022) and their diversity in Australian savannas is among the highest anywhere in the world (Andersen and Vasconcelos 2022). Furthermore, ants respond to variation in fire regimes, especially through fire-mediated modifications of habitat openness through changes in vegetation structure (Andersen 2019; Brassard et al. 2023, 2024). Fire also affects resource use by ants (Oliveira et al. 2024).

We base our analysis on comprehensive ant sampling of a long-term (18 years), replicated fire experiment with six fire treatments varying in fire frequency, seasonality and intensity (Brassard et al. 2024). We have previously described ant community responses to the fire frequency and seasonality treatments (hereafter referred as fire regime treatments) as well as to fire activity, a metric cumulating fire intensity over the course of the experiment (Brassard et al. 2023, 2024). The great majority of species are epigaeic and arid-adapted; their abundance and richness are promoted by frequent fire because of the open habitats maintained by it (Brassard et al. 2023). The arboreal ant fauna is dominated by epigaeic species and so is similarly favoured by frequent fire (Brassard et al. 2024). In contrast, subterranean ants have not responded to fire and the plot-level abundance and richness of cryptobiotic species in leaf litter are *highest* in the absence of fire, although almost all species were present in a wide range of fire treatments (Brassard et al. 2024). Building on our previous studies, here we use a biodiversity-maximisation approach to identify how many of the fire regime treatments or fire activity classes are necessary for representing ant diversity. We examine two measures of diversity: (1) species richness, as a basis for maximising species occurrence; and (2) geometric mean abundance (GMA), a metric sensitive to the abundance of species that is negatively correlated with extinction risk (Buckland et al. 2011; Kelly et al. 2014; McCarthy et al. 2014; Davies et al. 2018).

## Materials and methods

### Study system

Our study uses a long-term fire experiment at the Territory Wildlife Park (TWP; 12°42′S 130°59′E), located approximately 50 km south of Darwin in Australia’s Northern Territory. The climate is monsoonal, with almost all (ca. 90%) of the mean annual rainfall (1809 mm) falling from November to April (Bureau of Meteorology 2022). Mean monthly minimum and maximum temperatures are 20–25 °C and 31–33 °C, respectively (Bureau of Meteorology 2022). The vegetation of the study site is savanna woodland dominated by *Eucalyptus tetrodonta* and *E. miniata*, over a C4 grassy understorey (Scott et al. 2009). Such vegetation is typical of the high-rainfall (≥ 1000 mm per annum) regions of the Australian monsoonal tropics. The site has a highly diverse ant fauna, with over 150 (mostly undescribed) species (Parr and Andersen 2008; Brassard et al. 2023).

### Experimental fire regime treatments

**Fig. 1:**
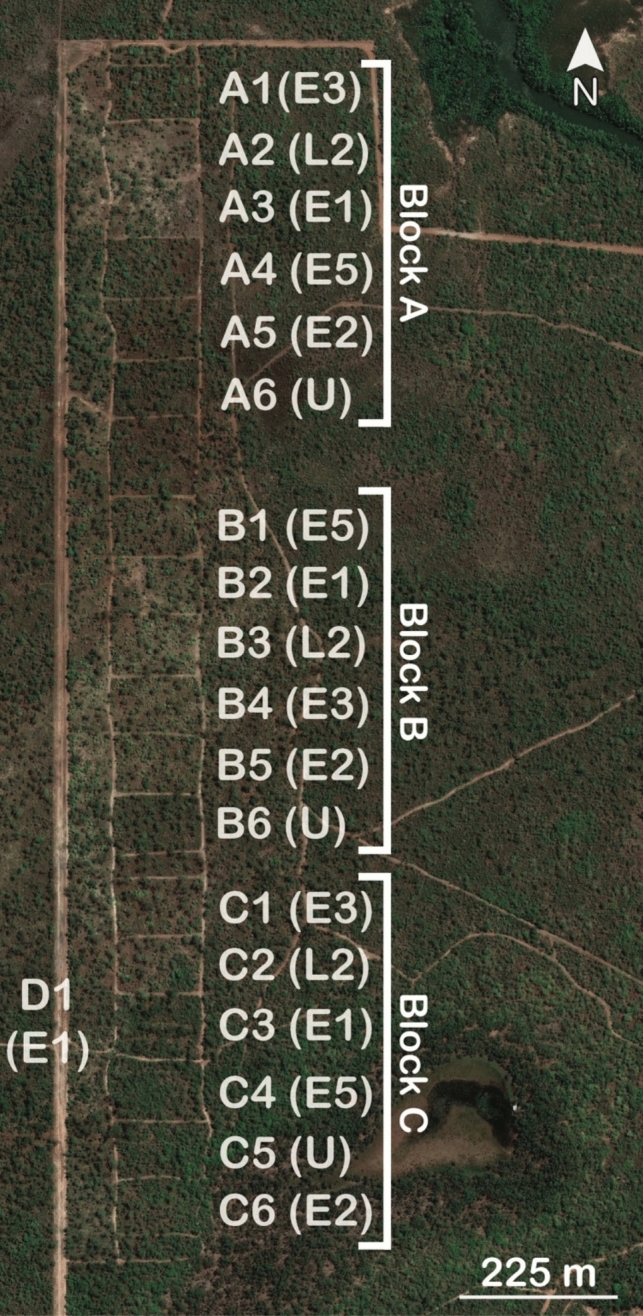
Map of all 1-ha plots (*n* = 18) at the Territory Wildlife Park fire experiment. Each block (i.e., **A**, **B** and **C**) consists of six plots receiving one of six fire regime treatments (shown in brackets): burnt early (first week of June) in the dry season every one (E1), two (E2), three (E3) and five (E5) years; burnt late (first week of October) in the dry season every 2 years (L2); and unburnt (U). The block A is furthest upslope and has relatively shallow soils and water availability, while the C block is furthest downslope, with deeper soils and higher soil water availability. Note that for this study we used a replacement (D1) for the E1 plot in the C block for fire treatment analyses because the original remained largely unburnt due to extremely sparse grass cover. D1 was placed in the eastern buffer zone which is annually burnt at the same period as the E1 treatment

**Fig. 2:**
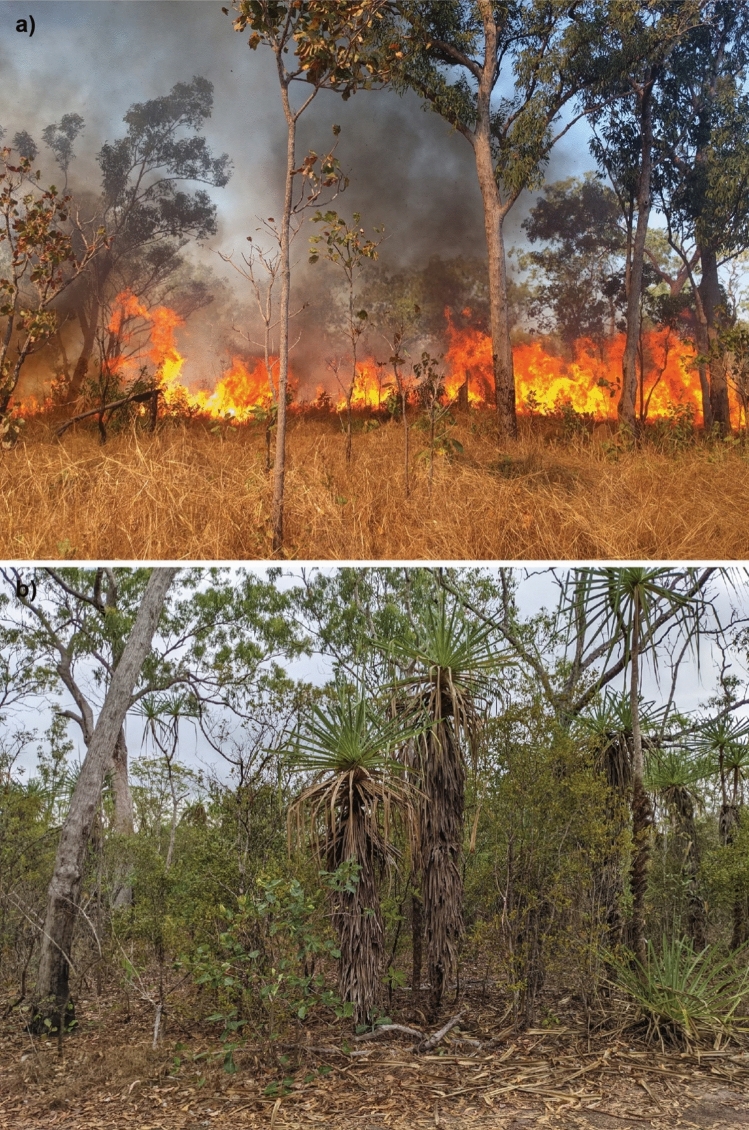
Plots receiving **a** higher fire frequency have lower woody cover than plots with **b** lower fire frequency. Photos by Alan Andersen

The experiment consists of 18 × 1-ha plots in a randomised block design, with six experimental fire regime treatments that differ primarily in frequency, and to a lesser extent seasonality, distributed in each of three blocks (A, B and C; see Fig. 1). The fire regime treatments commenced in 2004 and are: burnt early (first week of June; relatively low intensity: mean ranging between 546 and 847 kW m^−1^) in the dry season every 1 (E1), 2 (E2), 3 (E3) and 5 (E5) years; burnt late (first week of October; relatively high intensity: mean of 1184 kW m^−1^) in the dry season every 2 years (L2); and unburnt (U). For our analyses of fire regime treatments, we used a replacement E1 plot for the C block because the original, C3, remained largely unburnt due to extremely sparse grass cover. We situated this new plot (D1) adjacent to the plot it replaced, in a zone burnt every year at the same time as the E1 plots (Fig. 1). Prior to the establishment of experimental fire treatments, the area had been protected from fire since the early 1980s. Experimental plots receiving higher fire frequencies and biennial late dry season fires have lower woody cover (Levick et al. 2019)(Fig. 2).

### Fire activity classes

Over the 19-year history of the fire experiment, Byram’s fire-line intensity (rate of forward spread × fuel load × heat yield of fuel; with units; kilowatts per meter; Byram 1959) was estimated for each experimental fire. We used these fire intensity estimates to create a fire activity metric, which sums all the fire intensities a plot has received at the time of sampling (for more details see Brassard et al. 2023, 2024). Then, to make our fire activity analyses comparable with the fire regime treatments analyses, we separated plots in six fire activity classes, from 0 (no fire activity) to 5 (highest fire activity). We used the original C3 plot for these analyses because fire intensity was recorded in it, but not in the replacement D1 plot. As a result, the total species list slightly differs between our analyses of fire regime treatments and fire activity classes.

### Ant data

We combine data from three sampling events conducted between March 2021 and February 2022, which collected 133 ant species (Brassard et al. 2024). For each sampling event, we surveyed all plots (between 9:00 and 15:00), placing 15 epigaeic pitfall traps, 15 arboreal traps, 30 subterranean traps and doing 3 leaf litter extractions per plot. We pooled data from all sampling events to establish a list of species and their abundance (i.e., sum of trap occurrences for each species) for each plot. Pitfall traps were unbaited, whereas the subterranean (Eppendorf tubes placed 10 cm below ground) and arboreal (falcon tubes placed on tree trunks at approx. 130 cm height) were baited with an inside coating of a mixture of tuna, peanut butter, and honey. The traps were distributed among 15 sampling stations in a 5 × 3 grid with 10-m spacing near the centre of each plot. Each station had 1 epigaeic pitfall trap, 1 arboreal trap and two subterranean traps, all partially filled with ethylene glycol and deployed for 48 h. In each transect of the grid, we sifted 1 m^2^ of leaf litter in four locations, centred between each of the stations, then hung the leaf litter in mini-Winkler extractors for 72 h.

### Data analysis

#### Ant data

A total of 133 species were considered, but after removing species found in a single plot (and therefore likely an artefact of inherent spatial variation rather than reflecting fire preference) 98 species remained. We then removed the three exotic species (*Tetramorium lanuginosum*, *Tetramorium simillimum*, and *Trichomyrmex destructor*). We further excluded nine specialist forest species that are not part of the savanna fauna; these are all common and widespread in forest throughout the high rainfall regions of the Northern Territory and beyond and so are not of conservation concern. This left a total of 86 native savanna species to use for our fire regime treatment analyses. We followed the same approach for our fire activity classes analyses and were left with a total of 89 native savanna ant species.

#### GMA calculations

We calculated the geometric mean abundance using the formula:

$$\text{GMA}=\sqrt[n]{{x}_{1} {x}_{2} {x}_{3}\dots {x}_{n}}$$

where *n* represents the total number of species in the species pool, and *x* represent the abundance of a species. We used the number of traps a species was detected in a plot as our measure of abundance because ants are colonial organisms and counting individual workers could largely inflate abundance values for a species if a trap was placed near a colony. Since the calculation of GMA requires non-zero values, to include all 86 species in all plots we added a constant of 1 to all species abundances prior to the calculation (additive or Laplace smoothing), then subtracted 1 from the resulting GMA values. The value of the constant used in GMA calculations affects the influence of rare or absent species, with lower constant values heightening their influence. To verify that using a different constant value did not change our results substantially, we also produced a second set of analyses where we calculated GMA using a smaller constant of 0.1, which it did not (see Appendix, Fig. S1–S5). We used relatively small constant values for both calculations to avoid overshadowing variations in rare species across treatments and plots, especially since most species at our study sites are rarely collected.

#### Fire regime treatments and fire activity classes comparisons

We initially compared mean richness and GMA among fire regime treatments and fire activity classes using analyses of variances (ANOVAs).

We then identified all possible unique combinations of the six fire treatments, where each treatment could only be used once in a combination (i.e., no repetitions within a combination). For a given combination size *r* (ranging from 1 to 6), the total number of possible combinations is calculated as:

$$C\left(n,r\right)=\frac{n!}{r!\left(n-r\right)!}$$

where *n* is the total number of fire treatments (i.e., 6), and *r* is the number of treatments in the combination. Using this formula, the total number of unique combinations for *r* = 1 to *r* = 6 is:

- C(6,1) = 6C(6, 1) = 6;
- C(6,2) = 15C(6, 2) = 15;
- C(6,3) = 20C(6, 3) = 20;
- C(6,4) = 15C(6, 4) = 15;
- C(6,5) = 6C(6, 5) = 6; and
- C(6,6) = 1C(6, 6) = 1.

This results in a total of 63 unique combinations across all group sizes. For each unique combination, we calculated the number of fire treatments present (i.e., 1–6), which we termed ‘diversity of fire regime treatment’.

For each level of fire treatment diversity (i.e., 1–6) we calculated the minimum, maximum, and median species richness and GMA. To assess how richness and GMA values vary if fire treatments were selected randomly, we randomly selected treatment combinations for each level of fire treatment diversity a thousand times. We then selected the most common richness and GMA value for each level of treatment diversity, which we refer to as random richness and random GMA. For this analysis, we standardised GMA for each level of treatment diversity by dividing species abundances with the number of fire treatments used for that combination, thus comparing mean GMA for each combination. We produced species accumulation curves for scenarios minimising, maximising, averaging or randomising diversity accumulation as we increased the number of fire regime treatments. Our approach to maximising diversity is a simplified version of reserve selection algorithms prioritising areas for protection as nature reserves in that it uses biodiversity complementarity to maximise diversity (Sarkar 2012). However, here we use fire treatments instead of nature reserves, and do not take into consideration area, connectivity and the cost of protection/management. We repeated the same approach for fire activity classes.

We recognise that in having only three spatial replicates (i.e., plots) per fire regime treatment, the effects of combining treatments might be due to increasing the number of plots (and therefore sampling area) rather than adding heterogeneity. We assessed this by comparing: (1) the species richness and GMAs of pairs of plots that were either from the same fire regime treatment or different treatments; and (2) the species richness and GMAs of trios of plots with the same fire regime treatment, two different treatments or three different treatments. We repeated this approach using pairs of plots grouped to represent either contrasting fire frequencies (U and E1), contrasting fire intensity (U and L2) and similar fire frequencies and intensities (E3 and E2; E3 and E5). For these analyses, we used linear mixed models (LMMs) in the package *lme4* (Bates et al. 2014), using the identity of the first plot of each combination as a random effect to control for pseudo-replication (i.e., the repeated use of the same plot in different combinations).

We used a similar approach with our fire activity classes (i.e., levels ordered from 0 to 5). For contrasting pairs, we used 0 vs 5 and 1 vs 5 as *contrasting* fire activity classes, and 1 vs 2 and 2 vs 3 as *similar* fire activity classes.

All analyses were performed using *R* (R Core Team 2024) and all figures plotted using *ggplot2* (Wickham 2016).

## Results

### Mean richness and GMA

**Fig. 3:**
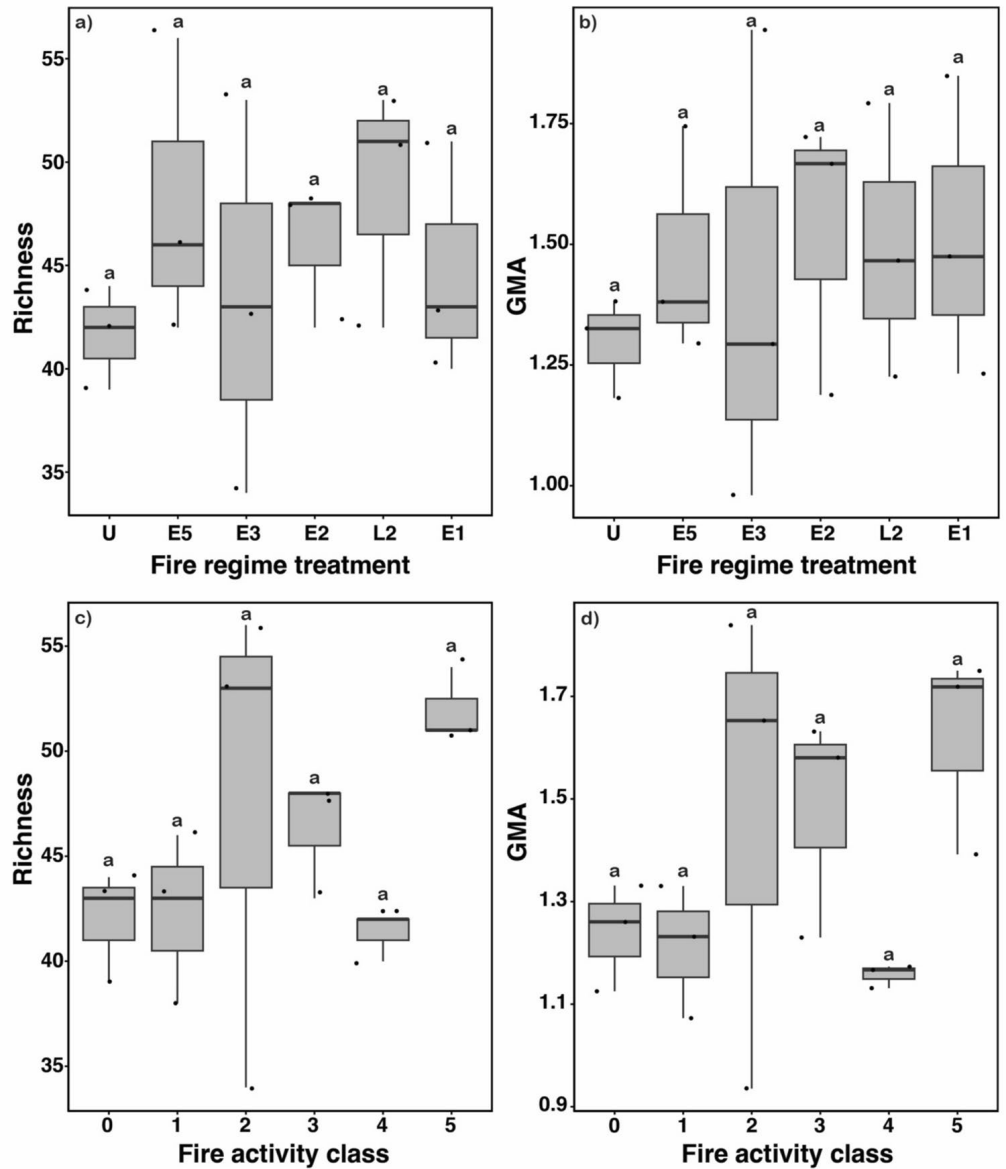
Variation in **a** richness and **b** GMA for fire regime treatments. Variation in **c** richness and **d** GMA for fire activity classes. Different letters indicate statistically different means. The grey box indicates the first and third quartiles (25th and 75th percentiles) and the horizontal line inside the box indicates the median. The whiskers indicate the largest value no further than 1.5 times the inter-quartile range from the upper and lower quartiles

Mean ant species richness per plot ranged from 41.7 in treatment U to 48.7 in treatment L2 (Table 1) but did not differ significantly among fire regime treatments (ANOVA, *F*_5,12_ = 0.58, *adjr*^*2*^ = −0.14, *p* = 0.72, Fig. 3a). The total species richness per fire regime treatment was similar (range: 68–72) for plots burnt every 1–3 years (E1, E2, L2 and E3). It was somewhat lower (65) for E5 and was markedly lower (52) for the unburnt treatment (Table 1). One species was recorded in only a single fire regime treatment (*Lioponera brevis* in E5, recorded in two of the three plots), whereas the 85 other species were recorded in multiple treatments.

**Table 1:** Mean (± standard error) species richness and GMA for plots of a fire regime treatment (*n* = 3) and total number of species and total GMA for each treatment

Fire regime treatment	Species richness		GMA	
	Mean	Total	Mean	Total
L2	48.7 ± 3.4	72	1.49 ± 0.16	4.51
E1	44.7 ± 3.3	68	1.52 ± 0.18	4.47
E2	46.0 ± 2.0	69	1.53 ± 0.17	4.79
E3	43.3 ± 5.5	70	1.41 ± 0.28	4.84
E5	48.0 ± 4.2	65	1.47 ± 0.14	4.32
U	41.7 ± 1.5	52	1.30 ± 0.06	3.2

The mean GMA for each plot ranged from 1.30 in the unburnt treatment to 1.53 in E2 (Table 1) and, as for species richness, did not differ significantly among fire regime treatments (ANOVA, *F*_5,12_ = 0.24, *adjr*^*2*^ = −0.29, *p* = 0.94, Fig. 3b). As for species richness, total GMA was markedly lower for the unburnt treatment compared to all other fire regime treatments (Table 1).

Mean species richness per plot ranged from 42 in the fire activity class ‘0’ to 52 in the fire activity class ‘5’ (Table 2). However, mean richness did not differ significantly between fire activity classes (ANOVA, *F*_5,12_ = 1.76, *adjr*^*2*^ = 0.42, *p* = 0.20, Fig. 3c). Total species richness per fire activity class (i.e., pooled across replicate plots) was lowest in the fire activity class ‘0’ (53), and highest in the fire activity classes ‘2’, ‘3’ and ‘5’ (range: 72–74) (Table 2). Only two species were recorded in a single fire activity class, *Pheidole* sp. (*variabilis* gp.) and *Pheidole* sp. 1 (*mjobergi* gp.), in the low and high fire activity classes, respectively.

**Table 2:** Mean (± standard error) species richness and GMA for plots of a fire activity class (*n* = 3) and total number of species and total GMA for each class

Fire activity class	Species richness		GMA	
	Mean	Total	Mean	Total
5	52.0 ± 1.0	72	1.62 ± 0.11	5.09
4	41.3 ± 0.7	60	1.16 ± 0.01	3.08
3	46.3 ± 1.7	74	1.48 ± 0.12	4.48
2	47.7 ± 6.9	73	1.48 ± 0.28	4.61
1	42.3 ± 2.3	59	1.21 ± 0.07	3.36
0	42.0 ± 1.5	53	1.24 ± 0.06	3.03

The mean GMA for each plot ranged from 1.16 in the fire activity class ‘4’ to 1.62 in the fire activity class ‘5’ (Table 2) but, as for species richness, did not differ significantly among fire classes (ANOVA, *F*_5,12_ = 1.82, *adjr*^*2*^ = 0.19, *p* = 0.18, Fig. 3d). Similar to fire regime treatments, the lowest total GMA was in the fire activity class ‘0’ (Table 2). The fire activity class ‘5’ had the highest total GMA (Table 2).

### Richness and GMA accumulation curves

**Fig. 4:**
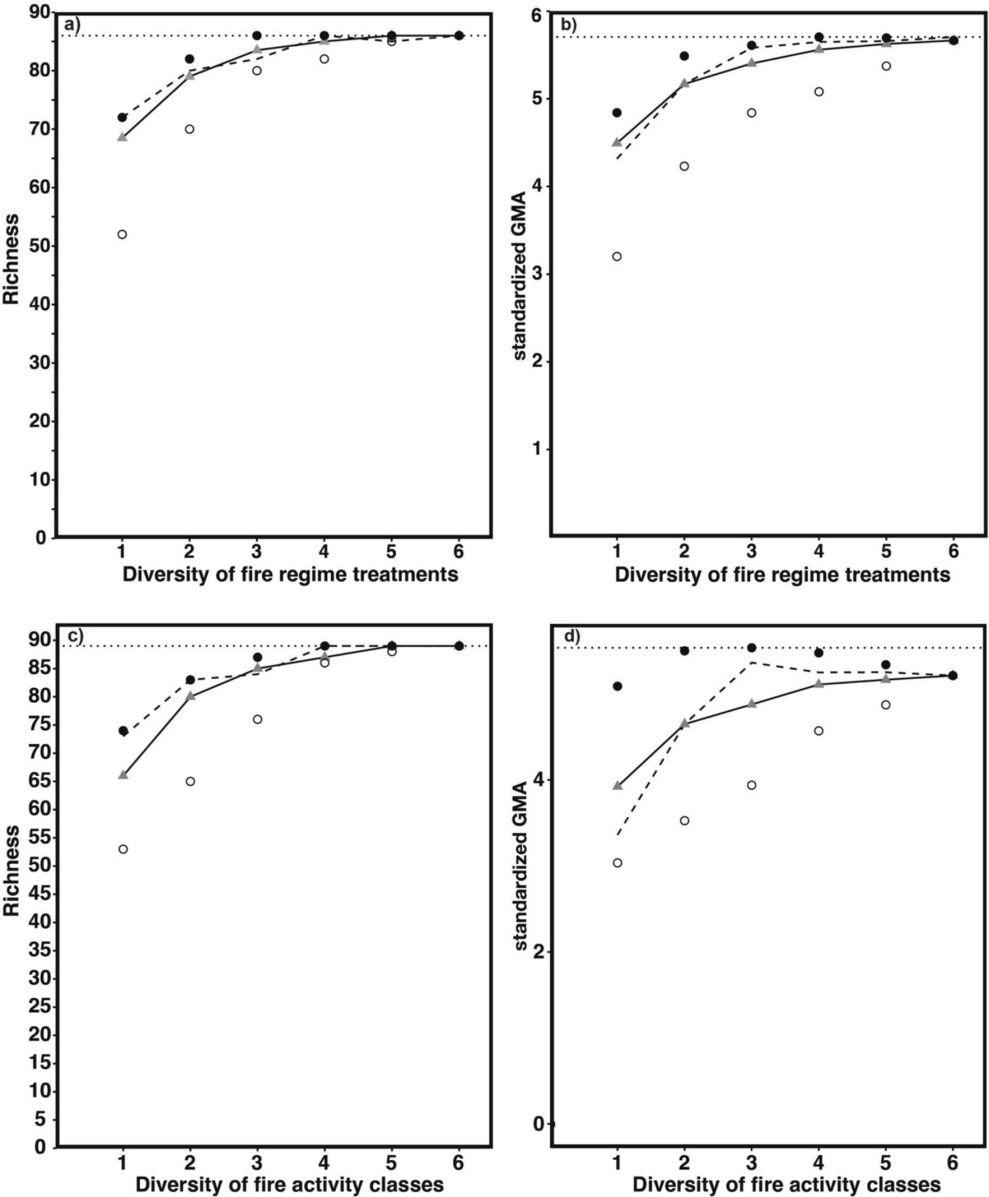
Accumulation curves showing the maximum (black points), the median (grey triangles) and the minimum (white points) **a** species richness and **b** standardised GMA obtained for each level of fire regime treatment diversity. The same is done for **c** species richness and **d** standardised GMA obtained for each level of fire activity class diversity. The dashed lines show the **a**–**c** richness **b**–**d** GMA accumulation curve when combinations were selected at random for each diversity level, as established by using the most common values after 1000 random selections. The solid lines follow the median. The horizontal dotted lines indicate the highest values

For combinations maximising species richness, only three fire regime treatments were necessary to include all species, whereas all six treatments were required if combinations minimising species richness were used (Fig. 4a). Median and random species richness also closely tracked maximum species richness for every number of fire regime treatments (Fig. 4a). For combinations maximising GMA, four fire regime treatments were necessary to reach the asymptote, whereas all six treatments were required when using combinations minimising GMA (Fig. 4b). Median and random GMA also closely tracked optimal GMA for every number of fire regime treatments (Fig. 4b).

For fire activity classes, four were necessary in combinations maximising species richness to include all species, whereas all six classes were necessary if combinations minimising species richness were used (Fig. 4c). Median and random species richness also closely tracked maximum species richness for every number of classes (Fig. 4c). For combinations maximising GMA, three classes were necessary to reach the asymptote, but then maximum GMA diminished by adding further fire activity classes (Fig. 4d). Contrary to richness, median and random GMA did not closely track optimal GMA fire activity classes (Fig. 4d).

### Richness and GMA of combinations of fire treatments and classes

**Fig. 5:**
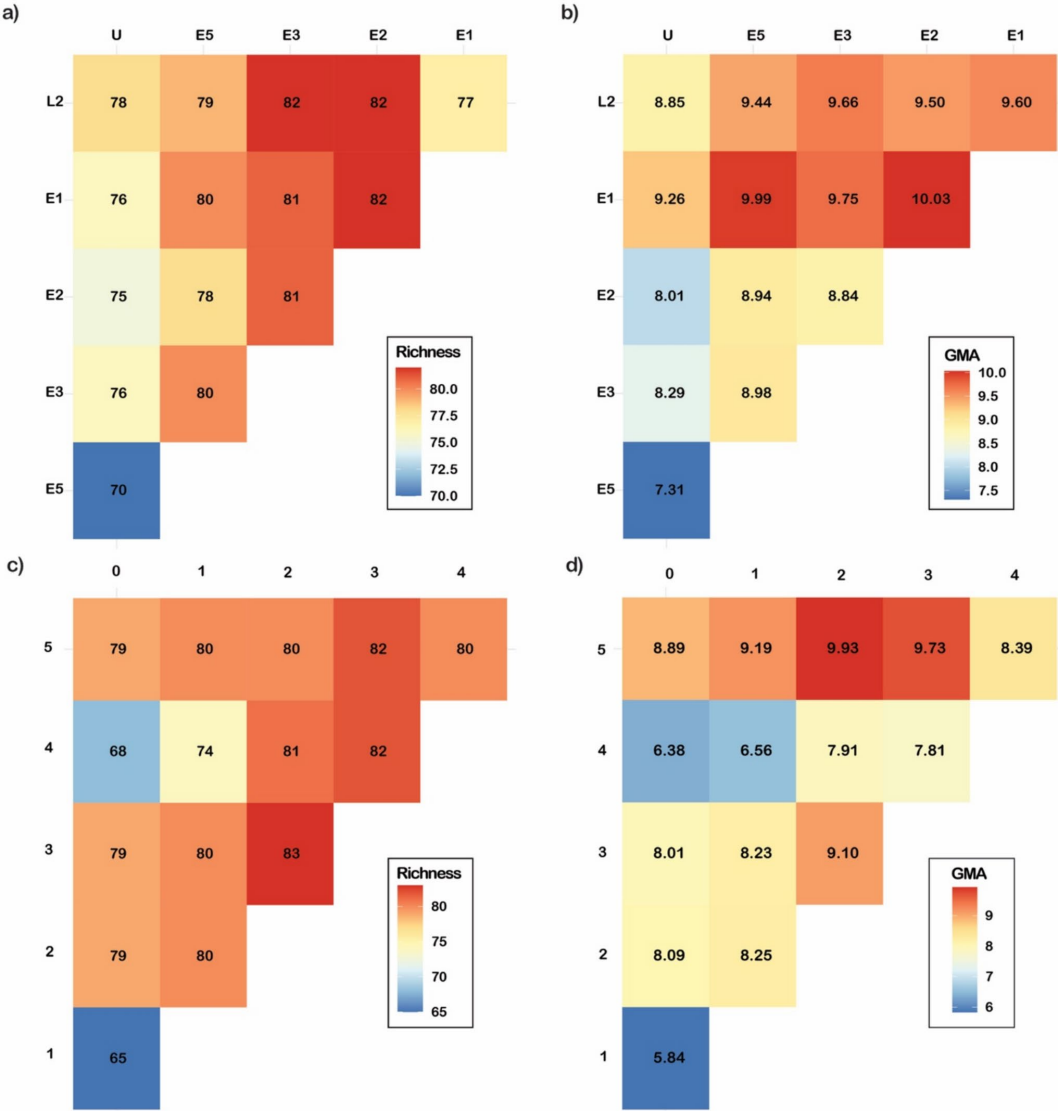
Heatmaps of **a** species richness and **b** GMA for every combination of two fire regime treatments. Heatmaps of **c** species richness and **d** GMA for every combination of two fire activity classes

Combinations of only two fire regime treatments represented 81.4% (E5 and U) to 95.3%, (E1 and E2; E2 and L2; E3 and L2) of total species (Fig. 5a). The four combinations with the lowest richness all included the unburnt treatment (Fig. 5a). Similarly, the three combinations with the lowest GMA all included the unburnt treatment (U and E2; U and E3; U and E5), whereas the three highest all included E1 (E1 and E2; E1 and E3; E1 and E5) (Fig. 5b). Combinations of three fire regime treatments represented 93–100% of the total species pool (Appendix, Table S2). The four combinations with lowest species richness all included the unburnt treatment (Appendix, Table S2). For three-treatment combinations covering at least 84 of the 86 total species, the most common fire regime treatments were E3 (7), E2 and E5 (both 6), and the least common was U (1).

Combinations of two fire activity classes represented 73% (‘0’ and ‘1’) to 93% (‘2’ and ‘3’) of total species (Fig. 5c). The three combinations with the lowest species richness included either the ‘0’ or ‘1’ fire activity classes, whereas the three combinations with the highest richness included the fire activity class ‘3’. Similarly, the three lowest values for GMA included either the fire activity classes ‘0’ or ‘1’ (Fig. 5d). In contrast, the three highest GMA values included the fire activity class ‘5’.

### Richness and GMA of pairs and trios of plots

At the plot level, the species richness of paired plots consisting of different fire regime treatments did not differ statistically from those consisting of the same treatment (LMM, *F*_1, 137_ = 0.15, *R2m* = 0, *R2c* = 0.46, *p* = 0.70, Fig. S6a). Similarly, richness did not differ between trios of plots of either the same fire regime treatment, two different treatments or all different treatments (LMM, *F*_2, 799_ = 1.25, *R2m* = 0, *R2c* = 0.46, *p* = 0.29 Fig. S6b). GMA of paired plots consisting of different fire regime treatments also did not differ statistically from that consisting of the same treatment (LMM, *F*_1, 137_ = 0.07, *R2m* = 0, *R2c* = 0.48, *p* = 0.79, Fig. S6c). Likewise, GMA did not differ between trios of plots of either the same fire regime treatment, two different treatments, or all different treatments (LMM, *F*_2, 800_ = 0.01, *R2m* = 0, *R2c* = 0.38, *p* = 0.99, Fig. S6d).

At the plot level, the species richness of paired plots consisting of different fire activity classes did not differ statistically from those consisting of the same class (LMM, *F*_1, 136_ = 2.29, *R2m* = 0, *R2c* = 0.53, *p* = 0.13, Fig. S6e). Richness did differ between trios of plots of either the same fire activity class, two different fire activity classes or all different fire activity classes, although the explanatory power of the relationship was nil (LMM, *F*_2, 798_ = 8.86, *R2m* = 0, *R2c* = 0.40, *p* = 1.42^–04^, Fig. S6f). Nevertheless, richness for trios of plots consisting of three different fire activity classes had higher richness on average than trios of plots with just one different fire activity classes (Tukey HSD: *p* = 3.00^–04^). GMA of paired plots consisting of different fire activity classes also did not differ statistically from that consisting of the same fire activity class (LMM, *F*_1, 137_ = 1.04, *R2m* = 0, *R2c* = 0.51, *p* = 0.31, Fig. S6g). As for richness, GMA did differ between trios of plots of either the same fire activity class, two different fire activity classes or all different fire activity classes, although the explanatory power of the relationship was also nil (LMM, *F*_2, 800_ = 5.84, *R2m* = 0, *R2c* = 0.39, *p* = 2.90^–03^ Fig. S6h). As for richness, GMA for trios of plots consisting of three different fire activity classes had higher values on average than trios of plots with just one different fire activity class (Tukey HSD: *p* = 4.5^–03^).

**Fig. 6:**
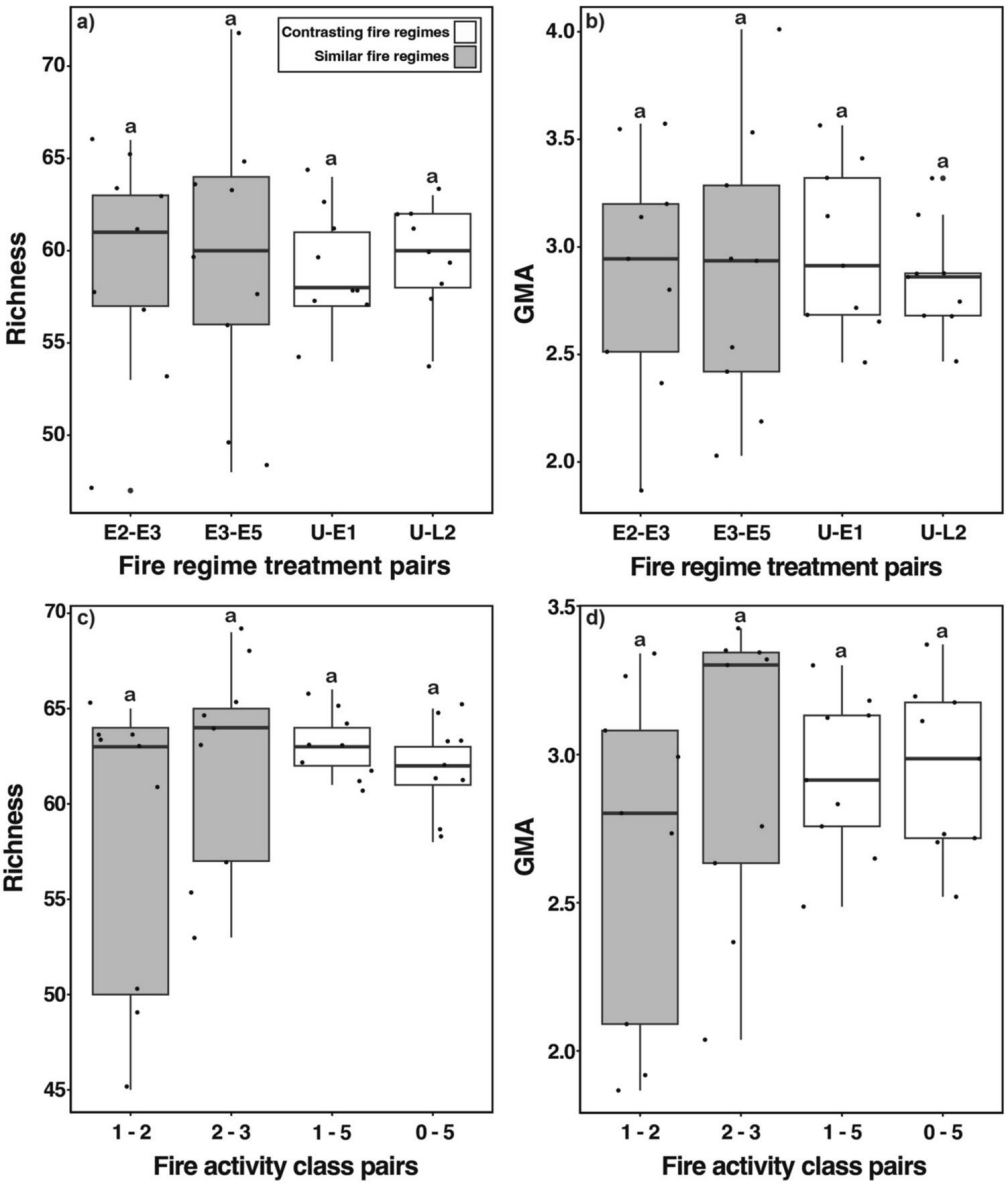
Comparison of **a** species richness and **b** GMA for pairs of plots from fire regime treatments of either similar fire frequencies and intensity (E2–E3 and E3–E5), pairs of plots from treatments of contrasting fire regime (U-E1) and pairs of plots from treatments of contrasting fire intensity (U-L2). Comparison of **c** species richness and **d** GMA for pairs of plots of similar fire activity classes (1–2 and 2–3) and pairs of plots of contrasting fire activity classes (1–5 and 0–5). Each point represents either the **a**–**c** richness or **b**–**d** GMA of one unique combination. Different letters indicate statistically different means. The box indicates the first and third quartiles (25th and 75th percentiles) and the horizontal line inside the box indicates the median. The whiskers indicate the largest value no further than 1.5 times the inter-quartile range from the upper and lower quartiles

At the plot-level, the species richness of paired plots consisting of contrasting fire regime treatments did not differ statistically from that consisting of similar treatments (LMM, *F*_1, 15_ = 0.69, *R2m* = 0, *R2c* = 0.49, *p* = 0.42, Fig. 6a). GMA of paired plots consisting of contrasting fire regime treatments did not differ statistically from that consisting of similar treatments (LMM, *F*_3, 22_ = 0.26, *R2m* = 0, *R2c* = 0.49, *p* = 0.85, Fig. 6b). Meanwhile, the species richness of paired plots consisting of contrasting fire activity classes also did not differ statistically from that consisting of similar fire activity classes (LMM, *F*_3, 23_ = 0.24, *R2m* = 0, *R2c* = 0.49, *p* = 0.88, Fig. 6c). Similarly, GMA of paired plots consisting of contrasting fire activity classes did not differ statistically from that consisting of similar fire activity classes (LMM, *F*_3, 22_ = 0.15, *R2m* = 0, *R2c* = 0.67, *p* = 0.70, Fig. 6d).

## Discussion

The pyrodiversity–biodiversity hypothesis has attracted extensive research attention over the past 20 years, but few studies have explicitly addressed the key question of how much pyrodiversity is required to conserve biodiversity. We tested the hypothesis for Australian tropical savanna ants, extending previous studies that describe biodiversity responses to fire (Parr and Andersen 2008; Andersen et al. 2014; Brassard et al. 2023, 2024), by explicitly addressing the key management question of how much pyrodiversity is required to maintain diversity. We do this through a novel application of the biodiversity-maximisation approach used for reserve selection in strategic conservation planning, based on data from a long-term, replicated field experiment. We found that a very limited number of fire regime treatments or fire activity classes are needed to represent most of the highly diverse savanna ant species and to maximise the geometric mean abundance of ants.

With just one exception for fire regime treatments and two for fire activity classes, all ant species were recorded across multiple fire treatments/classes. This demonstrates very low fidelity of species to specific fire regimes. Indeed, the three exceptions are likely a statistical artefact of rarity rather than true fire regime specialists.

Mean species richness and GMA at the plot level did not vary with fire regime treatment or fire activity class. However, the total diversity values tended to be lowest in treatments/classes receiving little or no fire, whereas the highest diversity tended to be in plots receiving frequent fire or high fire activity. This reinforces previous findings that fire promotes ant diversity in Australian tropical savannas (Andersen et al. 2014; Brassard et al. 2023).

Only two to three of the six treatments/classes were required to represent most or all species. Indeed, any two-treatment combination of burning every 1–3 years represented at least 94% of total species. In comparison, combinations involving the unburnt treatment gave at most 91% representation, and this fell to 81% when combined with the 5-yearly burning treatment. Similarly, the fire activity classes ‘0’ and ‘1’ recovered the least species and had the lowest GMA. The unburnt treatment, which is also the fire activity class ‘0’, therefore provided little complementarity to the other treatments and classes. This reinforces a previous finding that long-term fire exclusion provides no conservation benefit to the local ant fauna (Andersen and Hoffmann 2011). GMA also reached optimal values quickly, with three fire regime treatments nearly reaching the asymptote. Only two fire activity classes were enough to nearly reach the asymptote, and optimal GMA values even started decreasing by adding more classes. This indicates that the addition of more fire regime treatments and fire activity classes had no meaningful conservation benefit to maximise GMA.

For both richness and GMA, the median and randomised selections of fire regime treatments closely tracked the maximum, suggesting that treatment identity was less important than the addition of plots for increasing diversity. This was likewise the case for richness and fire activity classes, although median and randomised GMA were noticeably lower than the maximum GMA, which may have been driven by the high GMA of the fire activity class ‘5’. Our comparisons of cumulative species richness and GMA of combined plots from the same treatment/class with those from different treatments/classes showed no meaningful differences in their diversity. Furthermore, we found no differences even when we compared plots from contrasting fire treatments/classes. This strongly suggests that combining treatments/classes mostly increases species richness and GMA by increasing the area sampled rather than through fire heterogeneity. As such, it is possible that any of the fire regime treatment or fire activity class other than the unburnt would recover all species if additional plots were included.

**Fig. 7:**
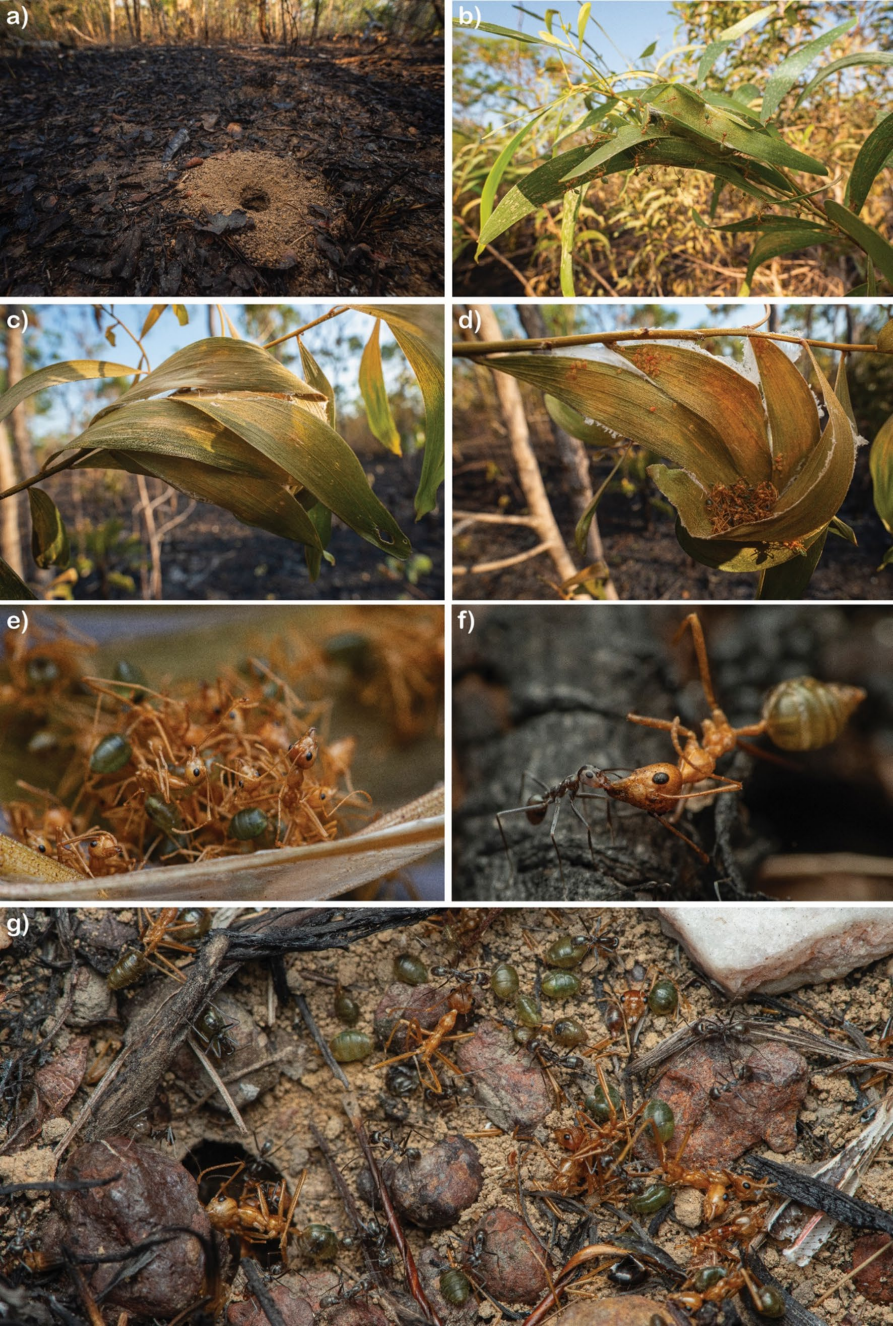
The species *O. smaragdina* is considered a ‘fire loser’ because their arboreal nests are vulnerable to fire. In contrast, epigaeic (i.e. ground-dwelling) species can escape fire by taking refuge underground inside their nest. Nevertheless, *O. smaragdina* persists even in plots receiving the highest fire activity because virtually every fire leaves a sufficient proportion of the vegetation within a plot unburned. Live nests of **a** ground-dwelling species and **b** *O. smaragdina* after an experimental fire. Nest of *O. smaragdina* that did not survive the fire **c**–**e**. A ground-dwelling species*, Iridomyrmex* sp. (*minor* gp.), took advantage of an *O. smaragdina* nest to take the dead workers back to their nest as fodder for their larvae **f**, **g**. Photos by François Brassard

Our finding that minimal pyrodiversity is required to conserve ant diversity in our study system does not imply that species respond uniformly to fire. Rather, it can be attributed to the substantial heterogeneity of fire behaviour and vegetation structure within individual fire regime treatments (Brassard et al. 2023), such that a wide variety of microhabitats is maintained within individual fire regimes. Given that ants have relatively small foraging ranges (Eguchi et al. 2004), only small patches of preferred microhabitats may be necessary for the persistence of a species. Thus, the scale of heterogeneity needed for biodiversity may be much smaller than for larger fauna. For instance, the green tree ant (*Oecophylla smaragdina*), despite being a known ‘fire loser’ (Andersen et al. 2007), persists in small unburnt enclaves within plots receiving the highest fire intensities (Fig. 7).

However, a single fire regime may not result in similar small scale burn heterogeneity in different ecosystems, or even within savannas of different biogeographic regions, especially since savannas of the Americas, Africa and Australia differ in their vegetation composition and trait diversity (Solbrig 1996; Simpson et al. 2022). There are clear intercontinental differences in large scale fire frequency and fire extent between these savannas (Simpson et al. 2022), but whether or not they also differ in their small-scale burning heterogeneity is unknown. Moreover, the biogeographic history of savanna ant faunas of different continents modulates their resilience to fire (Andersen and Vasconcelos 2022). As such, the levels of pyrodiversity needed to maintain ant diversity likely vary across savannas. We expect that, where a single fire regime leads to a high small scale burn heterogeneity such as in our system, low pyrodiversity will maintain high levels of biodiversity. In contrast, we expect that habitats where a single fire regime leads to low small-scale burn heterogeneity, higher levels of pyrodiversity will be required to maintain biodiversity. Future studies assessing how varying levels of small-scale burn heterogeneity affect ant diversity across bioregions will better our understanding of the mechanisms driving biodiversity responses to fire.

## Conclusion

Since natural fire intervals for tropical savannas in Australia are 2–4 years and the managed fire intervals are 1–3 years (Enright and Thomas 2008), there likely is no need to modify prevailing fire management to conserve ant diversity at the landscape scale. However, we acknowledge that our findings for ants cannot be generalised to all components of biodiversity. In particular, ant species are able to persist in small local patches in a way that taxa with larger home ranges cannot, such that the level of effective pyrodiversity within a single fire regime will vary widely among taxa. Notably, small mammals and birds are likely to require larger patch sizes, and they are far more sensitive to variation in fire regimes in Australian savannas than are ants (Andersen et al. 2012; Andersen 2021). We believe that our reserve selection approach to explicitly addressing how much pyrodiversity is required to maintain biodiversity has general applicability and we recommend its use in other fire-prone systems and for other taxa.

## Supplementary Information

Below is the link to the electronic supplementary material.

Supplementary file1 (DOCX 1555 KB)

## Data Availability

The data were deposited in Dryad as the following: Brassard, François; Murphy, Brett; Andersen, Alan (2024). Ant abundance in pitfall traps, subterranean traps, arboreal traps, and Winkler samples at the Territory Wildlife Park experiment [Dataset]. Dryad. 10.5061/dryad.08kprr57z

## Code availability

The datasets used and/or analysed during the current study are available from the corresponding author on reasonable request.
