# Supplementary material for: Large-scale pyrodiversity is not needed to beget ant diversity in an Australian tropical savanna
Source: Oecologia. 2025 Feb 28;207(3):41. doi: 10.1007/s00442-025-05683-7 (PMC11870973; doi:10.1007/s00442-025-05683-7)
Supplement: Supplementary file 1 — Supplementary file1 (DOCX 1555 KB) [file 442_2025_5683_MOESM1_ESM.docx]

**Large-scale pyrodiversity is not needed to beget ant diversity in an Australian tropical savanna**

François Brassard^1^*, Brett P. Murphy^1^, Simon Ferrier^2^, Alan N. Andersen^1^

^1^Charles Darwin University, Ellengowan Dr, Casuarina, Darwin NT 0810, Australia

^2^Commonwealth Scientific and Industrial Research Organisation, GPO Box 1700, Canberra, ACT 2601, Australia

*Corresponding author: François Brassard (francois.brassard.bio@gmail.com), Research Institute for the Environment & Livelihoods, Charles Darwin University, Ellengowan Drive, Casuarina, Darwin NT 0810, Australia

**Supplementary material**

**Appendix 1**

**Table S1** List of studies that assessed the pyrodiversity-biodiversity hypothesis. Note that this table extends on the synthesis table of Jones & Tingley (2022) by including more recent studies on the subject. Furthermore, this table compiles information on complementarity testing (i.e., if a study assessed what combination of fire regimes captured the most diversity).

| **reference** | **year** | **region** | **taxa** | **habitat** | **fire metric** | **faunal response** | **stat. approach** | **pyrodiversity-biodiversity supp.** | **complementarity test** |
| --- | --- | --- | --- | --- | --- | --- | --- | --- | --- |
| (Masters 1996) | 1996 | Australia | Reptiles (40 spp.) | Spinifex grassland | Time since fire | Abundance, richness | t-test | Yes | No |
| (Stuart-Smith et al. 2002) | 2002 | Canada | Birds | Boreal forest | Burned-unburned | Abundance, richness, log-alpha series index | Orthogonal contrasts | Yes | No |
| (Brotons et al. 2005) | 2005 | Spain | Birds | Mesic and xeric Mediterranean shrub and forest | Habitat category: forest or shrubland burned in the last 3-18 years | Richness, distribution, composition | ANOVA, Correspondence analysis (CA), GLMs, PCAs | Yes | No |
| (Cook and Holt 2006) | 2006 | North America | Beetles | Grasslands | Fire frequency | Richness, abundance, Shannon-Weaver diversity and evenness | ANOVAs, GLMs | No | No |
| (Parr and Andersen 2006) | 2006 | Australia & Africa | NA | NA | NA | NA | Review | No | No |
| (Andersen and Hoffmann 2011) | 2011 | Australia | Ants | Savanna | Fire frequency | Abundance, richness, composition | ANOVA, ANOSIM | No | No |
| (Pastro et al. 2011) | 2011 | Australia | plants, reptiles, mammals | Grasslands | Before after prescribed burn vs wildfire | Alpha and beta diversity | ANOVAs, ANOSIM | No | No |
| (Davies et al. 2012) | 2012 | South Africa | Insects: termites | savanna | Seasonality, frequency, precipitation interaction | Abundance, richness, composition | ANOVAs, Kruskal-Wallis, ANOSIM, IndVal | No | No |
| (Kelly et al. 2012) | 2012 | Australia | Small mammals | Mallee | Fire area and time since fire | Capture rate and species richness | GLMMs | No | No |
| (Langlands et al. 2012) | 2012 | Australia | Spiders | Grassland | Time since fire | Richness, evenness, composition | PERMANOVA | Yes | No |
| (Taylor et al. 2012) | 2012 | Australia | Birds | Mallee | Post-fire age classes | Richness | GLMs | No | No |
| (Nimmo et al. 2013) | 2013 | Australia | Reptiles | Mallee | post fire age class | Probability of Occurrence | GLMs | No | No |
| (Andersen et al. 2014) | 2014 | Australia | Ants | Savanna | Fire frequency/seasonality | Abundance, richness, composition | Linear mixed models, ANOSIM | No | No |
| (Farnsworth et al. 2014) | 2014 | Australia | Reptiles | Mallee | Time since fire age classes | Alpha, beta, gamma diversity | GLMMs | No | No |
| (Maravalhas and Vasconcelos 2014) | 2014 | Brazil | Ants | Savanna | Fire frequency, fire seasonality | Richness, composition | Multiple regression, PERMANOVAs, hierarchical diversity partitioning | Yes | No |
| (Sitters et al. 2014) | 2014 | Australia | Birds | Woodlands | Post-fire age classes | Richness, species turnover | GLMMs | Yes | No |
| (Kelly et al. 2014) | 2015 | Australia | Reptiles, small mammals, birds | Mallee | Time since fire classes | Probability of occurrence, geometric mean abundance (GMA) | GAMMs & SDMs | No | No |
| (Avitabile et al. 2015) | 2015 | Australia | Termites | Mallee | Time since fire | frequency of occurrence, richness | GAMMs, GLMMs | No | No |
| (Cohn et al. 2015) | 2015 | Australia | Plants | Forest/woodlands | Post fire age classes and vegetation type | Richness, species turnover, Simpson’s index and measure of eveness, Sorensen dissimilarity index | GLMs | Yes | No |
| (Lawes et al. 2015) | 2015 | Australia | Small mammals | Savanna | Fire extent (fire size and fire frequency) | Richness, abundance, GMA | GLMs | No | No |
| (Radford et al. 2015) | 2015 | Australia | Mammals | Savanna | Time since fire, fire frequency, fire seasonality, fire area | Abundance | GLMs | No | No |
| (Burgess and Maron 2016) | 2016 | Australia | Birds | Forest/Woodland | Fire history category | Richness | CAPs, GLMs, GLMMs | No | No |
| (Ponisio et al. 2016) | 2016 | North America | Bees, plants | Forest, woodland | Fire history metric incorporating frequency, age, extent and severity | Beta diversity, bee and plant richness, interaction richness | GLMs | Yes | No |
| (Tingley et al. 2016) | 2016 | North America | Birds | Forest, woodlands | Fire severity, time since fire | Occupancy, richness | Bayesian community model, multi-species hierarchical occupancy model | Yes | No |
| (Paolucci et al. 2016) | 2016 | Brazil | Ants | Forests | Fire treatments: unburned, burned in natural conditions, burned with 50% fuel addition | Seed removal, abundance, richness, composition | GLMMs, PERMANOVA, PERMDISP | No | No |
| (Anjos et al. 2017) | 2017 | Brazil | Ants | Grasslands | Unburnt and burnt fire history | Richness, abundance, composition | Rarefaction curves, ANOVAs | No | No |
| (Brown and York 2017) | 2017 | Australia | Flies, wasps | Heaty woodland | Fire history heterogeneity (time since fire & fire frequency) | Richness | Akaike;s information criterion corrected for small sample sice (AICc), Canonical Correspondence Analysis (CCA) | Yes | No |
| (Prowse et al. 2017) | 2017 | Australia | Birds | Woodlands | Time since fire | Abundance | GAMMs | No | No |
| (Paolucci et al. 2017) | 2017 | Brazil | Ants | Forests | Fire frequency | Abundance, richness, composition | GLMs, PERMANOVAs | No | No |
| (Beale et al. 2018) | 2018 | Africa | Birds, mammals | Savanna | Time since fire, fire area, seasonality, fire intensity | Richness | Integrated Nested Laplace Approximation | Yes | No |
| (Bliege Bird et al. 2018) | 2018 | Australia | Mammals (5 spp.), monitor lizards | Desert grassland | Fire history | Activity | Track plot surveys and satellite imagery, GLM | Yes | No |
| (Davies et al. 2018) | 2018 | Australia | Mammals | Savanna | Fire frequency | Abundance, geometric mean abundance (GMA) | GLMs | No | No |
| (McGranahan et al. 2018) | 2018 | North America | Plants | Grasslands | Fire frequency, seasonality, size | Biomass, functional diversity, beta diversity | Random-effects regression, multivariate dispersion of beta diversity, break point analysis, ordinary least squares regression | Yes | No |
| (Taillie et al. 2018) | 2018 | North America | Birds | Forest, woodlands | Fire severity, time since fire | Richness, abundance | Hierarchical distance sampling models | Yes | No |
| (Steel et al. 2019) | 2019 | North America | Bats | Forest, woodlands | Fire severity | Species occurrence, richness | Occupancy models | Yes | No |
| (Docherty et al. 2020) | 2020 | Africa | Birds | Savanna | Four measures of pyrodiversity & Spatial extent of fire age classes | Richness, functional richness, funct. Evenness, funct. Dispersion | LMMS, null models | Yes | No |
| (Wills et al. 2020) | 2020 | Australia | Birds | Forest, woodland | Post-fire age classes | Richness, composition | Kruskal-Wallis, t-tests, PERMANOVA | No | No |
| (Bishop et al. 2021) | 2021 | Greece | Ants | Forests, maquis | Burnt & unburnt fire treatments, canopy openness | Abundance, richness, composition, morphological traits | ANOVA, PERMANOVA, kernel density estimation | Yes | No |
| (Senior et al. 2021) | 2021 | Australia | Mammals: Microbats, macropods, echidnas, rodents | Mallee | Time since fire, area of time since fire age class, diversity of age class | Likelihood of occurrence | SDMs | Yes | No |
| (Gordijn and O’Connor 2021)) | 2021 | South Africa | plants | grassland | fire frequency, fire size | alpha, beta diversity and functional diversity | multivariate and Bayesian models | yes (partial support) | no |
| (López-Baucells et al. 2021) | 2021 | Mediterranean | Bats | shrublands, pine forests | fire recurrence | foraging activity | Maximum entropy models | No | No |
| (Jones and Tingley 2022) | 2022 | Worldwide (review) + Case study (North America) | Birds: spotted owl, black-backed woodpecker | Forest | Post-fire landscape heterogeneity | Habitat occurrence. | Literature review. | Yes | No |
| (Jorge et al. 2022) | 2022 | USA | Birds | Pine savanna | Time since fire | Richness, occupancy | hierarchical Bayesian multispecies occupancy models | Yes | No |
| (Ulyshen et al. 2022) | 2022 | USA | Pollinators (bees & butterflies) | Forest | Burn history, fire frequency, fire area, canopy openness | Abundance, richness and Shannon diversity | GLMs | Yes | No |
| (Brassard et al. 2023) | 2023 | Australia | Ants | Savanna | Fire frequency, fire intensity, canopy cover | Abundance, richness, composition | GLMMs, PERMANOVAs | No | No |
| (Davies et al. 2023) | 2023 | Australia | Mammals | Savanna | Fire size | species-level population size | Simulation models | No | No |

**Table S2** Total species richness for all possible fire regime treatment combinations. E1-5 – burnt in the early dry season every 1-5 years respectively; L2 – burnt late in the dry season every 2 years; U – unburnt.

| Fire regime treatment  combination | Species  richness |
| --- | --- |
| U | 52 |
| E5 | 65 |
| E3 | 70 |
| E2 | 69 |
| L2 | 72 |
| E1 | 68 |
| E5, U | 70 |
| E2, U | 75 |
| E1, U | 76 |
| E3, U | 76 |
| E1, L2 | 77 |
| E2, E5 | 78 |
| L2, U | 78 |
| L2, E5 | 79 |
| E1, E5 | 80 |
| E3, E5 | 80 |
| E1, E3 | 81 |
| E2, E3 | 81 |
| E1, E2 | 82 |
| E2, L2 | 82 |
| L2, E3 | 82 |
| E1, L2, U | 80 |
| E1, E5, U | 81 |
| E2, E5, U | 81 |
| L2, E5, U | 81 |
| E1, L2, E5 | 82 |
| E2, E3, U | 82 |
| E3, E5, U | 82 |
| E1, E2, U | 83 |
| E1, L2, E3 | 83 |
| E2, L2, U | 83 |
| E1, E2, L2 | 84 |
| E1, E2, E3 | 84 |
| E1, E2, E5 | 84 |
| E2, L2, E3 | 84 |
| E2, E3, E5 | 84 |
| L2, E3, U | 84 |
| E1, E3, E5 | 85 |
| E1, E3, U | 85 |
| E2, L2, E5 | 85 |
| L2, E3, E5 | 86 |
| E1, L2, E5, U | 82 |
| E1, E2, L2, U | 84 |
| E2, L2, E3, U | 84 |
| E1, E2, L2, E3 | 85 |
| E1, E2, E3, E5 | 85 |
| E1, E2, E3, U | 85 |
| E1, E2, E5, U | 85 |
| E1, L2, E3, U | 85 |
| E2, E3, E5, U | 85 |
| E1, E2, L2, E5 | 86 |
| E1, L2, E3, E5 | 86 |
| E1, E3, E5, U | 86 |
| E2, L2, E3, E5 | 86 |
| E2, L2, E5, U | 86 |
| L2, E3, E5, U | 86 |
| E1, E2, L2, E3, U | 85 |
| E1, E2, L2, E3, E5 | 86 |
| E1, E2, L2, E5, U | 86 |
| E1, E2, E3, E5, U | 86 |
| E1, L2, E3, E5, U | 86 |
| E2, L2, E3, E5, U | 86 |
| E1, E2, L2, E3, E5, U | 86 |
|  |  |


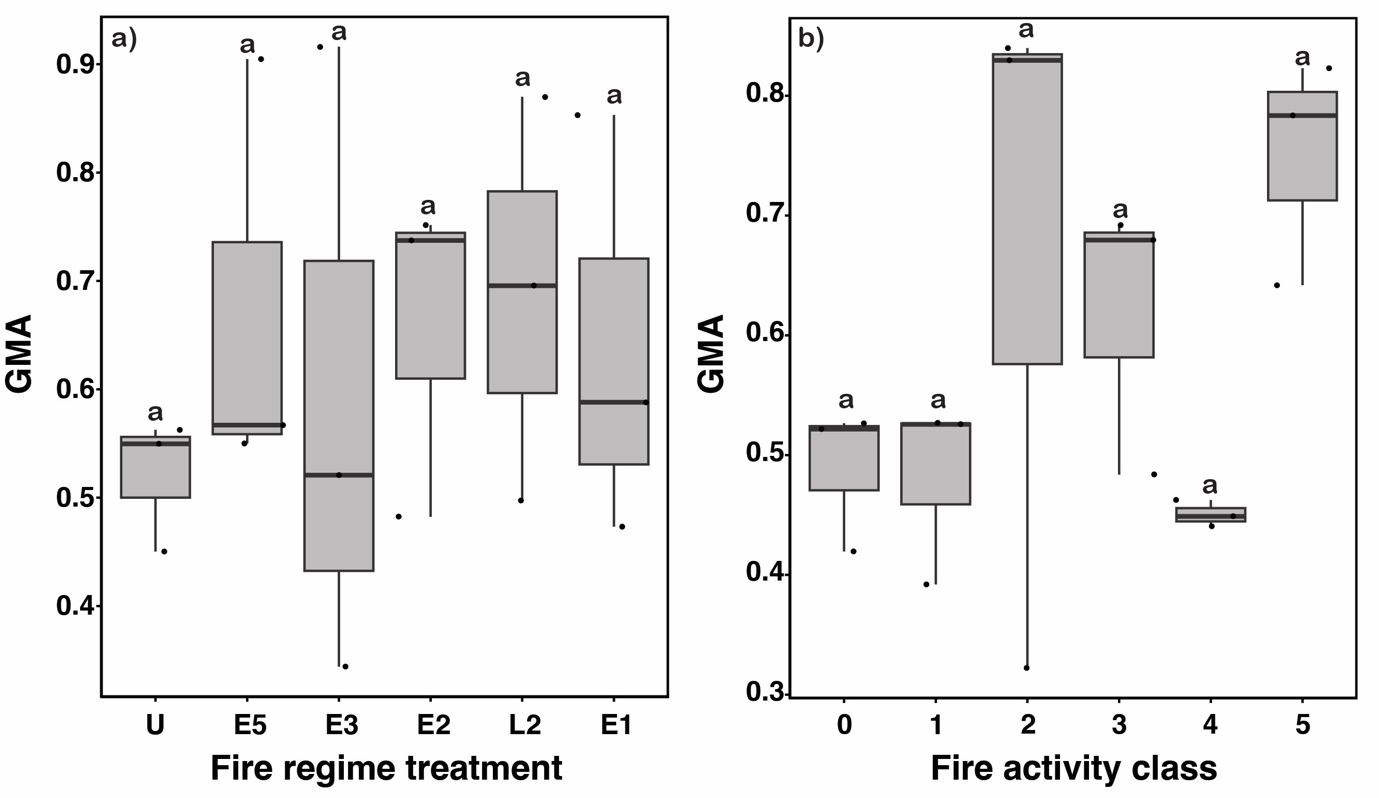


**Figure S1** Variation in GMA (calculated using a constant of 0.1 instead of 1) for a) fire regime treatments and b) fire activity classes. Different letters indicate statistically different means. The grey box indicates the first and third quartiles (25th and 75th percentiles) and the horizontal line inside the box indicates the median. The whiskers indicate the largest value no further than 1.5 times the inter-quartile range from the upper and lower quartiles.

**
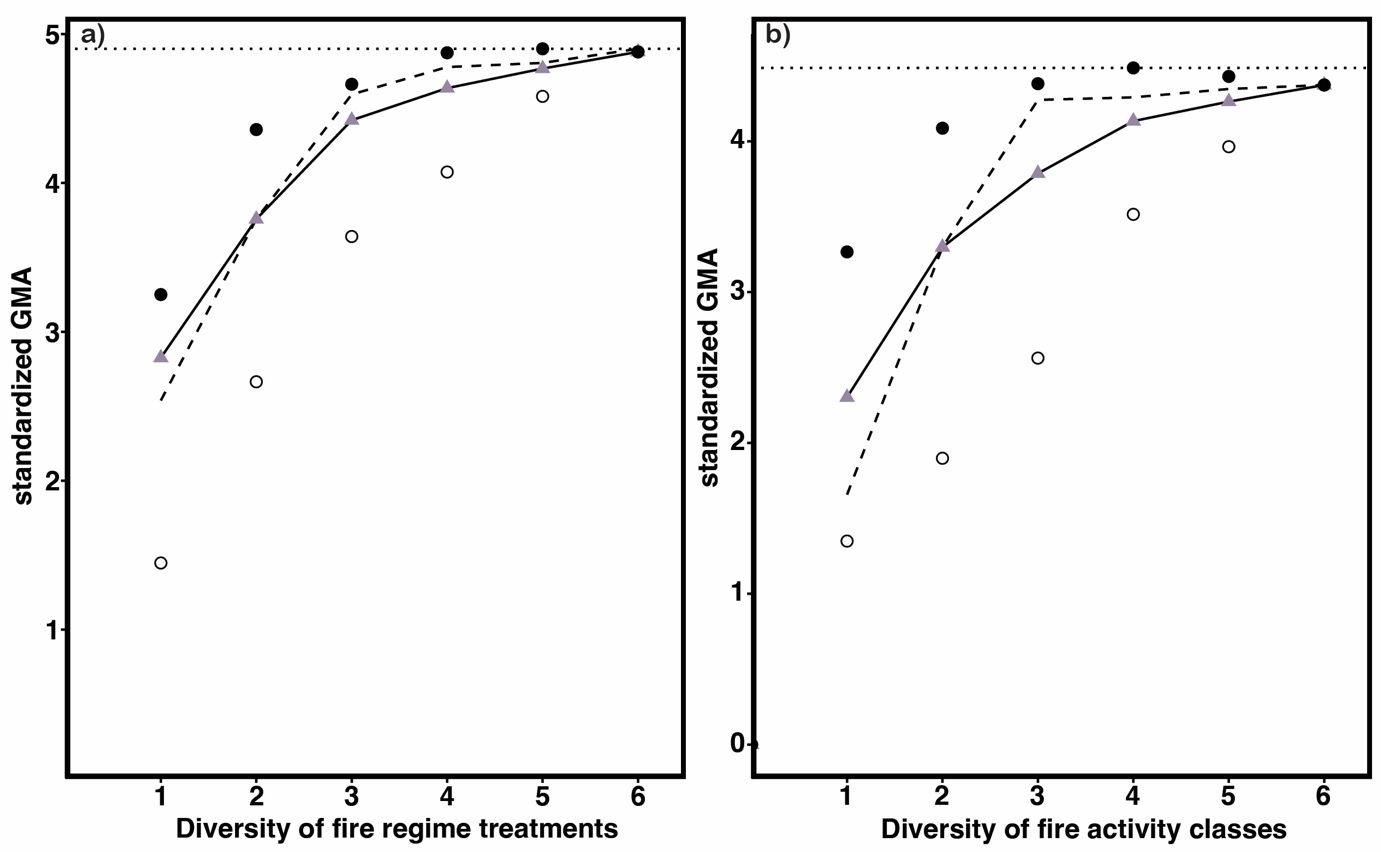
**

**Figure S2** Accumulation curves showing the maximum (black points), the median (grey triangles) and the minimum (white points) standardized GMA (calculated using a constant of 0.1 instead of 1) obtained with a) fire regime treatment or b) fire activity class combinations. The dashed lines show GMA accumulation curve when combinations were selected at random for each number group, as established by using the most common values after 1000 random selections. The solid lines follow the median. The horizontal dotted lines indicate the highest values.

**
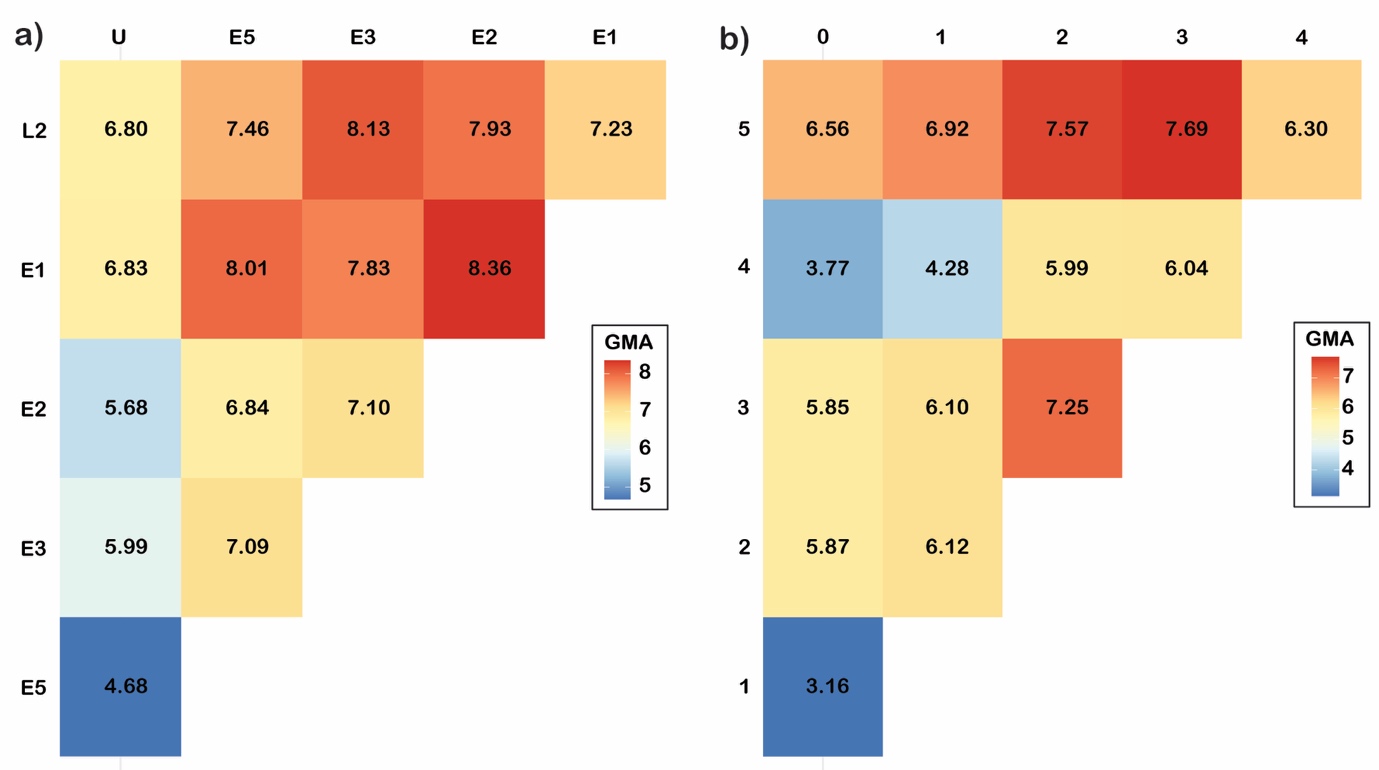
**

**Figure S3** Heatmaps of GMA (calculated using a constant of 0.1 instead of 1) for every combination of a) two fire regime treatments and b) two fire activity classes.


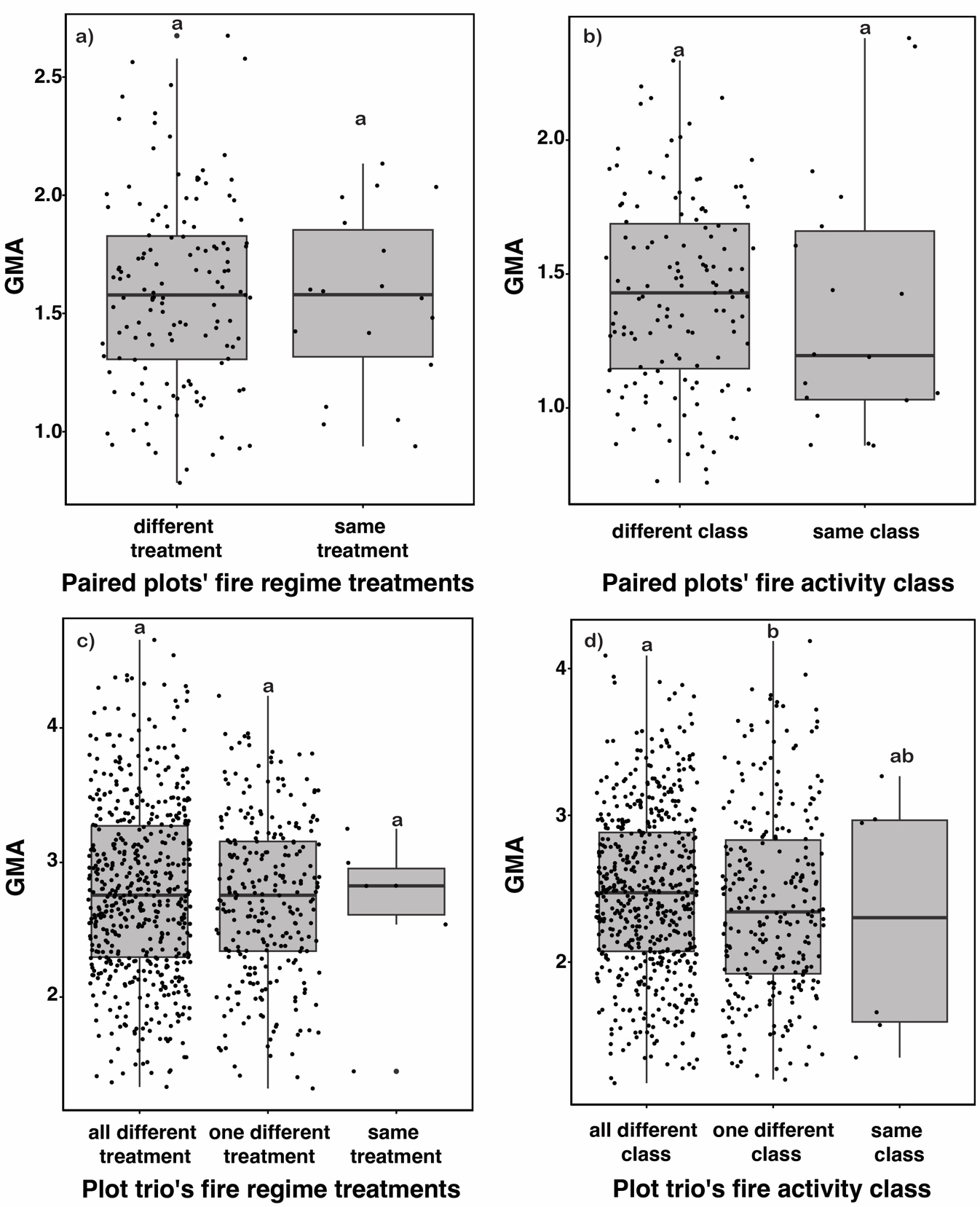


**Figure S4** Comparison of GMA (calculated using a constant of 0.1 instead of 1) for a) pairs of plots from either different fire regime treatments or the same treatment and b) pairs of plots from either different fire activity classes or the same class. Comparison of c) trios of plots consisting of either all different fire regime treatments, two plots of the same treatment plus one of a different treatment or all of the same treatment. In d), we repeat the same approach using fire activity classes. Each point represents the GMA of one unique combination. Different letters indicate statistically different means. The grey box indicates the first and third quartiles (25th and 75th percentiles) and the horizontal line inside the box indicates the median. The whiskers indicate the largest value no further than 1.5 times the inter-quartile range from the upper and lower quartiles.


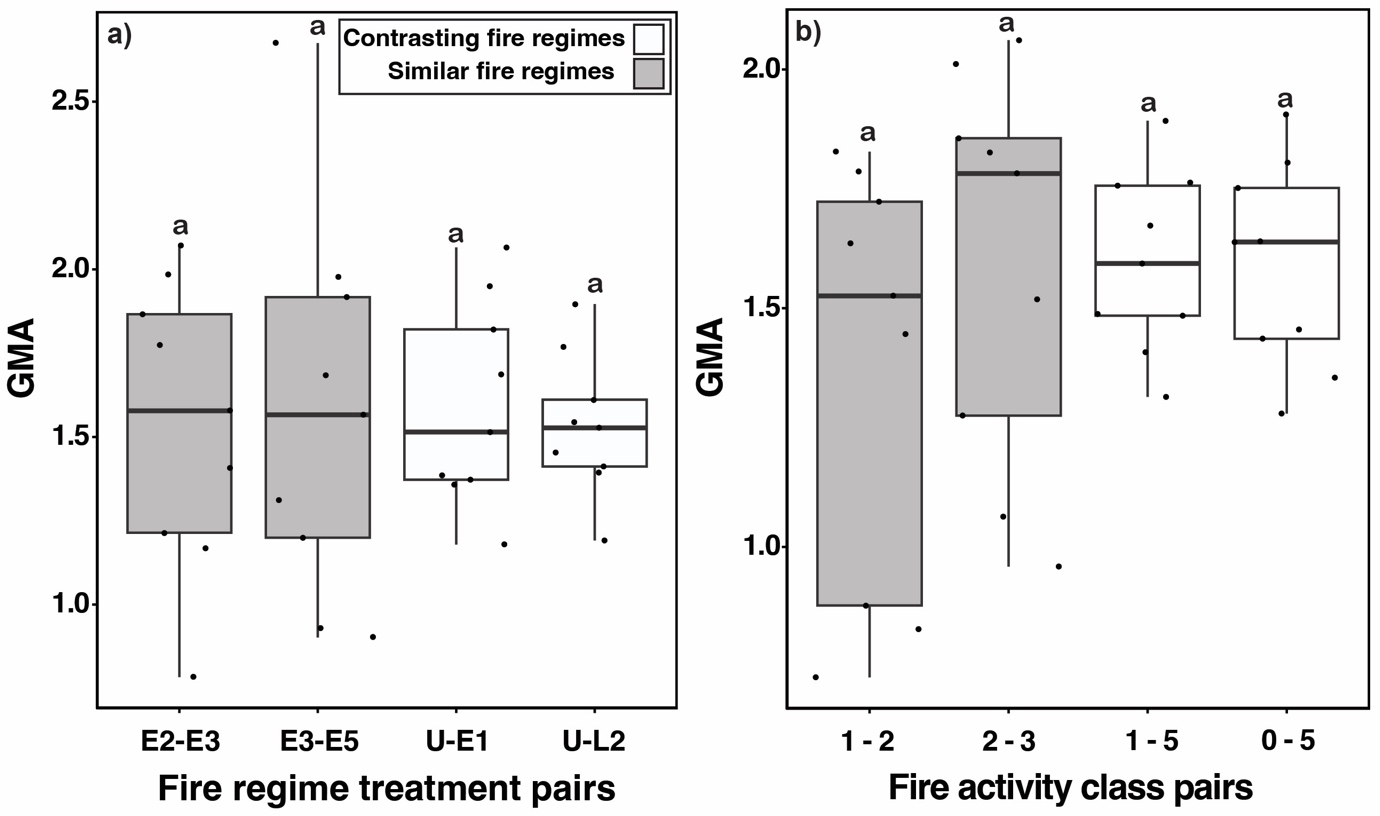


**Figure S5** Comparison of GMA (calculated using a constant of 0.1 instead of 1) for a) for pairs of plots from treatments of either similar fire regimes and intensity (E2-E3 and E3-E5), pairs of plots from treatments of contrasting fire regimes (U-E1) and pairs of plots from treatments of contrasting fire intensity (U-L2). Comparison of GMA for b) pairs of plots of similar fire activity classes (1-2 and 2-3) and pairs of plots of contrasting fire activity classes (1-5 and 0-5). Each point represents the GMA of one unique combination. Different letters indicate statistically different means. The box indicates the first and third quartiles (25th and 75th percentiles) and the horizontal line inside the box indicates the median. The whiskers indicate the largest value no further than 1.5 times the inter-quartile range from the upper and lower quartiles.

**
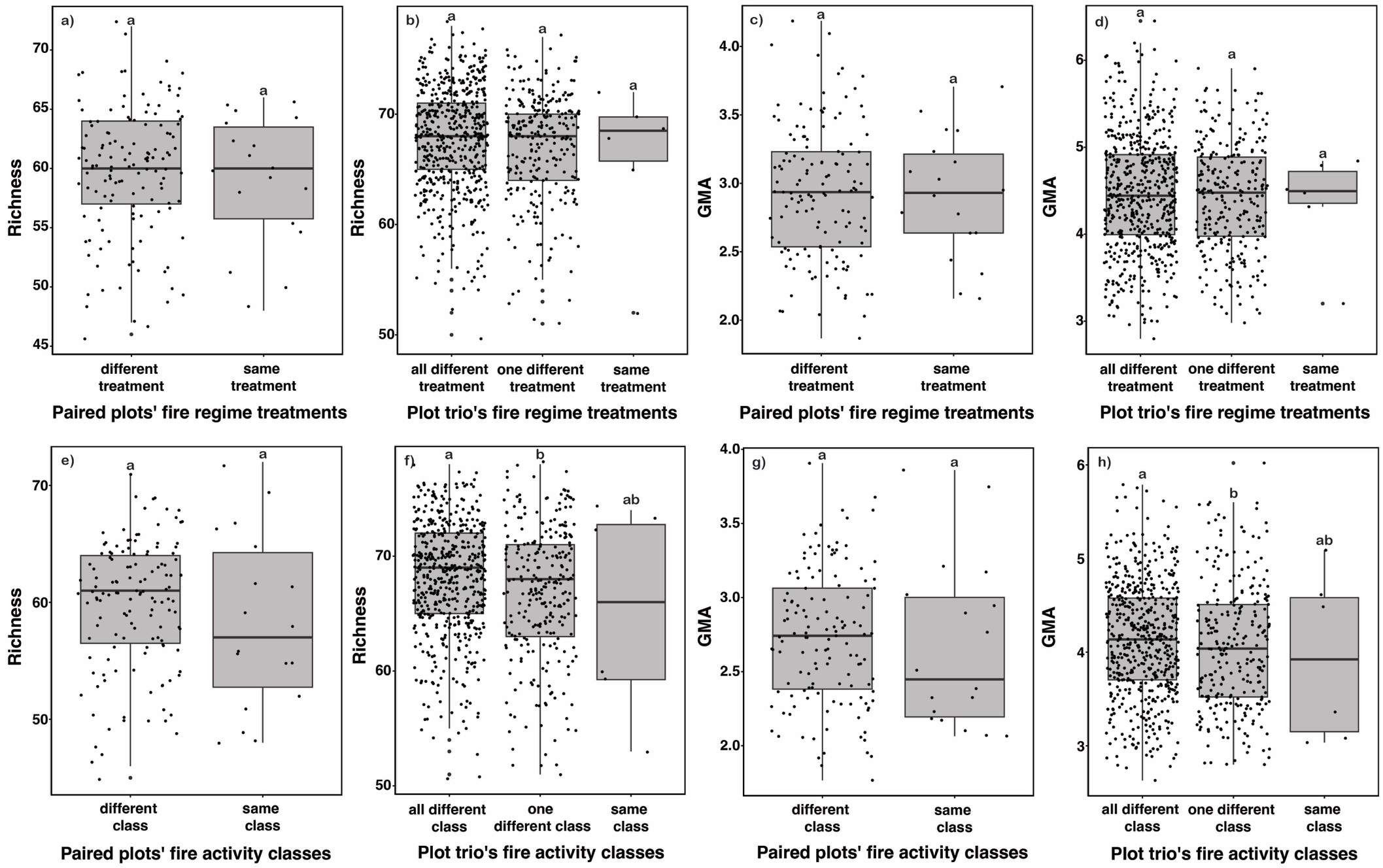
**

**Figure S6** Comparison of richness for a) pairs of plots from either different fire regime treatments or the same treatment and b) trios of all plots consisting of either two plots of the same treatment plus one of a different treatment or all of the same treatment. Comparison of GMA for c) pairs of plots from either different treatments or the same treatment and d) trios of plots consisting of either all different treatments, two plots of the same treatment plus one of a different treatment or all of the same treatment. We make the same comparisons, but for fire activity classes in e–h). Each point represents either the richness (a–b, e–f) or GMA (c–d, g–h) of one unique combination. Different letters indicate statistically different means. The grey box indicates the first and third quartiles (25th and 75th percentiles) and the horizontal line inside the box indicates the median. The whiskers indicate the largest value no further than 1.5 times the inter-quartile range from the upper and lower quartiles.

**References**

Andersen A, Hoffmann BD (2011) Conservation value of low fire frequency in tropical savannas: Ants in monsoonal northern Australia. Austral Ecol 36:497–503

Andersen A, Ribbons RR, Pettit M, Parr CL (2014) Burning for biodiversity: Highly resilient ant communities respond only to strongly contrasting fire regimes in Australia’s seasonal tropics. J Appl Ecol. https://doi.org/10.1111/1365-2664.12307

Anjos D, Campos R, Campos R, Ribeiro S (2017) Monitoring effect of fire on ant assemblages in brazilian rupestrian grasslands: Contrasting effects on ground and arboreal fauna. Insects 8:. https://doi.org/10.3390/insects8030064

Avitabile SC, Nimmo DG, Bennett AF, Clarke MF (2015) Termites are resistant to the effects of fire at multiple spatial scales. PLoS One 10:. https://doi.org/10.1371/journal.pone.0140114

Beale CM, Courtney Mustaphi CJ, Morrison TA, et al (2018) Pyrodiversity interacts with rainfall to increase bird and mammal richness in African savannas. Ecol. Lett.

Bishop TR, Tomlinson A, McNeice T, et al (2021) The effect of fire on ant assemblages does not depend on habitat openness but does select for large, gracile predators. Ecosphere 12:. https://doi.org/10.1002/ecs2.3549

Bliege Bird R, Bird DW, Fernandez LE, et al (2018) Aboriginal burning promotes fine-scale pyrodiversity and native predators in Australia’s Western Desert. Biol Conserv 219:. https://doi.org/10.1016/j.biocon.2018.01.008

Brassard F, Pettit MJ, Murphy BP, Andersen AN (2023) Fire influences ant diversity by modifying vegetation structure in an Australian tropical savanna. Ecology 1–15. https://doi.org/10.1002/ecy.4143

Brotons L, Herrando S, Martin J-L (2005) Bird assemblages in forest fragments within Mediterranean mosaics created by wild fires. Landsc Ecol 19:. https://doi.org/10.1007/s10980-005-0165-2

Brown J, York A (2017) Fly and wasp diversity responds to elements of both the visible and invisible fire mosaic. Int J Wildl Fire 26:. https://doi.org/10.1071/WF16189

Burgess EE, Maron M (2016) Does the response of bird assemblages to fire mosaic properties vary among spatial scales and foraging guilds? Landsc Ecol 31:. https://doi.org/10.1007/s10980-015-0275-4

Cohn JS, Di Stefano J, Christie F, et al (2015) How do heterogeneity in vegetation types and post-fire age-classes contribute to plant diversity at the landscape scale? For Ecol Manage 346:. https://doi.org/10.1016/j.foreco.2015.02.023

Cook WM, Holt RD (2006) Fire frequency and mosaic burning effects on a tallgrass prairie ground beetle assemblage. Biodivers Conserv 15:. https://doi.org/10.1007/s10531-004-8227-3

Davies AB, Eggleton P, Van Rensburg BJ, Parr CL (2012) The pyrodiversity-biodiversity hypothesis: A test with savanna termite assemblages. J Appl Ecol 49:422–430. https://doi.org/10.1111/j.1365-2664.2012.02107.x

Davies HF, McCarthy MA, Rioli W, et al (2018) An experimental test of whether pyrodiversity promotes mammal diversity in a northern Australian savanna. J Appl Ecol 55:. https://doi.org/10.1111/1365-2664.13170

Davies HF, Visintin C, Murphy BP, et al (2023) Pyrodiversity trade-offs: A simulation study of the effects of fire size and dispersal ability on native mammal populations in northern Australian savannas. Biol Conserv 282:. https://doi.org/10.1016/j.biocon.2023.110077

Docherty TDS, Hethcoat MG, MacTavish LM, et al (2020) Burning savanna for avian species richness and functional diversity. Ecol Appl 30:. https://doi.org/10.1002/eap.2091

Farnsworth LM, Nimmo DG, Kelly LT, et al (2014) Does pyrodiversity beget alpha, beta or gamma diversity? A case study using reptiles from semi‐arid Australia. Divers Distrib 20:663–673

Gordijn PJ, O’Connor TG (2021) Multidecadal effects of fire in a grassland biodiversity hotspot: Does pyrodiversity enhance plant diversity? Ecol Appl 31:. https://doi.org/10.1002/eap.2391

Jones GM, Tingley MW (2022) Pyrodiversity and biodiversity: A history, synthesis, and outlook. Divers. Distrib. 28

Jorge MH, Conner LM, Garrison EP, Cherry MJ (2022) Avian species richness in a frequently burned ecosystem: a link between pyrodiversity and biodiversity. Landsc Ecol 37:. https://doi.org/10.1007/s10980-022-01399-8

Kelly LT, Bennett AF, Clarke MF, Mccarthy MA (2014) Optimal fire histories for biodiversity conservation. Conserv Biol 29:. https://doi.org/10.1111/cobi.12384

Kelly LT, Nimmo DG, Spence-Bailey LM, et al (2012) Managing fire mosaics for small mammal conservation: A landscape perspective. J Appl Ecol 49:. https://doi.org/10.1111/j.1365-2664.2012.02124.x

Langlands PR, Brennan KEC, Ward B (2012) Is the reassembly of an arid spider assemblage following fire deterministic? Austral Ecol 37:. https://doi.org/10.1111/j.1442-9993.2011.02299.x

Lawes MJ, Murphy BP, Fisher A, et al (2015) Small mammals decline with increasing fire extent in northern Australia: Evidence from long-Term monitoring in Kakadu National Park. Int J Wildl Fire. https://doi.org/10.1071/WF14163

López-Baucells A, Flaquer C, Mas M, et al (2021) Recurring fires in Mediterranean habitats and their impact on bats. Biodivers Conserv 30:. https://doi.org/10.1007/s10531-020-02095-2

Maravalhas J, Vasconcelos HL (2014) Revisiting the pyrodiversity-biodiversity hypothesis: Long-term fire regimes and the structure of ant communities in a Neotropical savanna hotspot. J Appl Ecol. https://doi.org/10.1111/1365-2664.12338

Masters P (1996) The effects of fire-driven succession on reptiles in spinifex grasslands at Uluru national park, Northern Territory. Wildl Res 23:. https://doi.org/10.1071/WR9960039

McGranahan DA, Hovick TJ, Elmore RD, et al (2018) Moderate patchiness optimizes heterogeneity, stability, and beta diversity in mesic grassland. Ecol Evol 8:. https://doi.org/10.1002/ece3.4081

Nimmo DG, Kelly LT, Spence-Bailey LM, et al (2013) Fire Mosaics and Reptile Conservation in a Fire-Prone Region. Conserv Biol 27:. https://doi.org/10.1111/j.1523-1739.2012.01958.x

Paolucci LN, Maia MLB, Solar RRC, et al (2016) Fire in the Amazon: impact of experimental fuel addition on responses of ants and their interactions with myrmecochorous seeds. Oecologia 182:. https://doi.org/10.1007/s00442-016-3638-x

Paolucci LN, Schoereder JH, Brando PM, Andersen A (2017) Fire-induced forest transition to derived savannas: Cascading effects on ant communities. Biol Conserv 214:. https://doi.org/10.1016/j.biocon.2017.08.020

Parr CL, Andersen AN (2006) Patch Mosaic Burning for Biodiversity Conservation : a Critique of the Pyrodiversity Paradigm. Conserv Biol 20:1610–1619. https://doi.org/10.1111/j.1523-1739.2006.00492.x

Pastro LA, Dickman CR, Letnic M (2011) Burning for biodiversity or burning biodiversity? Prescribed burn vs. wildfire impacts on plants, lizards, and mammals. Ecol Appl 21:. https://doi.org/10.1890/10-2351.1

Ponisio LC, Wilkin K, M’Gonigle LK, et al (2016) Pyrodiversity begets plant-pollinator community diversity. Glob Chang Biol. https://doi.org/10.1111/gcb.13236

Prowse TAA, Collard SJ, Blackwood A, et al (2017) Prescribed burning impacts avian diversity and disadvantages woodland-specialist birds unless long-unburnt habitat is retained. Biol Conserv 215:. https://doi.org/10.1016/j.biocon.2017.09.005

Radford IJ, Gibson LA, Corey B, et al (2015) Influence of fire mosaics, habitat characteristics and cattle disturbance on mammals in fire-prone savanna landscapes of the northern Kimberley. PLoS One 10:. https://doi.org/10.1371/journal.pone.0130721

Senior KL, Giljohann KM, McCarthy MA, et al (2021) Predicting mammal responses to pyrodiversity: From microbats to macropods. Biol Conserv 256:. https://doi.org/10.1016/j.biocon.2021.109031

Sitters H, Christie FJ, Di Stefano J, et al (2014) Avian responses to the diversity and configuration of fire age classes and vegetation types across a rainfall gradient. For Ecol Manage 318:. https://doi.org/10.1016/j.foreco.2014.01.009

Steel ZL, Campos B, Frick WF, et al (2019) The effects of wildfire severity and pyrodiversity on bat occupancy and diversity in fire-suppressed forests. Sci Rep 9:. https://doi.org/10.1038/s41598-019-52875-2

Stuart-Smith K, Adams IT, Larsen KW (2002) Songbird communities in a pyrogenic habitat mosaic. Int J Wildl Fire 11:. https://doi.org/10.1071/WF01050

Taillie PJ, Burnett RD, Roberts LJ, et al (2018) Interacting and non-linear avian responses to mixed-severity wildfire and time since fire. Ecosphere 9:. https://doi.org/10.1002/ecs2.2291

Taylor RS, Watson SJ, Nimmo DG, et al (2012) Landscape-scale effects of fire on bird assemblages: Does pyrodiversity beget biodiversity? Divers Distrib 18:. https://doi.org/10.1111/j.1472-4642.2011.00842.x

Tingley MW, Ruiz-Gutiérrez V, Wilkerson RL, et al (2016) Pyrodiversity promotes avian diversity over the decade following forest fire. Proc R Soc B Biol Sci 283:. https://doi.org/10.1098/rspb.2016.1703

Ulyshen MD, Hiers JK, Pokswinksi SM, Fair C (2022) Pyrodiversity promotes pollinator diversity in a fire-adapted landscape. Front Ecol Environ 20:. https://doi.org/10.1002/fee.2436

Wills AJ, Liddelow G, Tunsell V (2020) Wildfire and fire mosaic effects on bird species richness and community composition in south-western Australia. Fire Ecol 16:1–15
